# Beef Consumption and Cardiovascular Disease Risk Factors: A Systematic Review and Meta-analysis of Randomized Controlled Trials

**DOI:** 10.1016/j.cdnut.2024.104500

**Published:** 2024-11-02

**Authors:** Lisa M Sanders, Orsolya M Palacios, Meredith L Wilcox, Kevin C Maki

**Affiliations:** 1 Midwest Biomedical Research, Addison, IL, United States; 2 Department of Applied Health Science, School of Public Health, Indiana University, Bloomington, IN, United States

**Keywords:** beef, red meat, cardiometabolic, cholesterol, triglyceride, systematic review, meta-analysis, randomized controlled trial

## Abstract

**Background:**

Results from observational studies suggest associations of red meat intake with increased risk of cardiovascular disease (CVD); however, RCTs have not clearly demonstrated a link between red meat consumption and CVD risk factors. Further, the specific effects of beef, the most consumed red meat in the United States, have not been extensively investigated.

**Objectives:**

This study aimed to perform a systematic review and meta-analysis of RCT data evaluating the effects of minimally or unprocessed beef intake on CVD risk factors in adults.

**Methods:**

A search of the literature was conducted using PubMed and CENTRAL databases. RCTs in adults that provided diets with fresh or minimally processed beef were included. Data were extracted, and pooled estimates from random-effects models were expressed as standardized mean differences (SMDs) between the beef intervention and comparator intervention with less or no beef. Sensitivity and subgroup analyses were also performed.

**Results:**

Twenty relevant RCTs that met the criteria were included. Beef intake did not impact blood pressure or most lipoprotein-related variables, including total cholesterol, HDL-cholesterol, triglycerides, non–HDL-cholesterol, apolipoprotein A or B, and VLDL-cholesterol. Beef consumption had a small but significant effect on LDL-cholesterol (0.11; 95% CI: 0.008, 0.20; *P* = 0.03), corresponding to ∼2.7 mg/dL higher LDL-cholesterol in diets containing more beef than that in low-beef or -o beef comparator diets. Sensitivity analyses show this effect was lost when 1 influential study was removed.

**Conclusions:**

Daily unprocessed beef intake do not significantly affect most blood lipids, apolipoproteins, or blood pressures, except for a small increase in LDL-cholesterol compared with diets with less or no beef. Thus, there may be other factors influencing the association of red meat and beef on CVD risk that deserve further investigation.

This study was registered at INPLASY as 202420013.

## Introduction

Red meat collectively refers to beef, goat, lamb, pork, veal, and game meats [1], and these are termed red meat due to their higher myoglobin content, which provides these meats with a deeper pink or red hue [2]. Although these meats vary in animal source and nutritional composition, they are frequently clustered together in studies assessing the effects of dietary components and/or patterns on cardiometabolic outcomes. As a result, generalizations of their effects on cardiometabolic health are collectively attributed to all red meat. Specifically, higher red meat intake has been associated with adverse cardiovascular disease (CVD) outcomes including CVD mortality in the United States [3,4] and risk of total stroke and ischemic stroke [5], although not all studies report an association [6,7]. The authors of a recent prospective study concluded that modeled replacement of 0.5 servings/d of red meat with 0.5 servings/d of nuts, whole grains, or skimmed milk was associated with 14%, 7% and 4% lower estimated risks for CVD, respectively [8].

Dietary patterns lower in red meat intake, such as a Mediterranean-style diet, have been associated with favorable effects on CVD markers such as triglycerides (TG), total cholesterol (TC), LDL-cholesterol, HDL-cholesterol, and systolic and diastolic blood pressures [9]. However, the authors of a 2022 meta-analysis assessing the effect of red meat intake on serum lipids and inflammatory markers concluded that red meat intake increased serum TG but had no effect on TC, LDL-cholesterol, HDL-cholesterol, C-reactive protein, and high-sensitivity C-reactive protein [10]. In addition, the authors of a 2019 meta-analysis concluded that the effects of red meat intake on CVD risk factors, including TC, LDL-cholesterol, HDL-cholesterol, and TG, was inconclusive compared with combined comparator diets (e.g., plant protein-based, chicken-based, fish-based, poultry-based, mixed protein–based, and carbohydrate-based diets) [11].

Further confounding the effect of clustering all red meat into a singular group when assessing its effects on cardiometabolic disease risk factors is that a number of dietary studies take an additional step and aggregate the already-collective red meat with processed meat, a type of meat product that is defined by its preparation, for example, curing, salting, and/or the addition of chemical preservatives such as nitrates [12]. Processed meat can be either white meat, for example, chicken, duck, and fish, and/or red meat in origin [12]. Findings from some studies indicate that processed red meat intake is associated with greater risk of CVD than unprocessed red meat [3,8].

Some authoritative bodies and health organizations recommend dietary patterns lower in red and processed meats [1,13], although the most recent Dietary Guidelines for Americans noted that dietary patterns higher in lean meats (which could include lean cuts of red meat) are associated with positive health outcomes [1]. Additionally, the American Heart Association and the National Lipid Association both recommend dietary patterns that allow for lean meat intake [14,15]. However, the American Heart Association still penalizes red meat intake, even lean, unprocessed varieties, in the dietary component of “Life’s Essential 8”, a metric designed to assess an individual’s or a population’s cardiovascular health [13].

Beef is the most frequently consumed type of red meat in the United States and, as a source of high-quality protein, zinc, iron, and vitamin B-12, could contribute to diet quality, particularly lean unprocessed beef. In fact, An et al. [16] reported that consumption of beef is associated with greater intake of protein, B vitamins, iron and zinc but is also associated with higher saturated fat intake. Lean, unprocessed beef is often used as a source of red meat in clinical trials evaluating CVD risk factors, and these studies often report no or little effects on CVD risk factors. Yet, the data from these trials have not been systematically reviewed as has been done with other red meats, such as pork [[17], [18], [19]]. Therefore, the objective of this investigation was to perform a systematic review and meta-analysis of results from RCTs evaluating the effects of fresh, unprocessed beef intake on selected CVD risk factors, specifically lipoprotein-related variables and systolic and diastolic blood pressures, and to assess whether the observed effects differ by study quality.

## Methods

### Literature search

This systematic review and meta-analysis followed the guidelines of PRISMA [20]. Potentially relevant articles were identified through a literature search using PubMed and CENTRAL databases through January 2024. The search criteria were designed to identify RCTs that evaluated the impact of beef intake on the CVD risk factors of lipoprotein-related variables and blood pressures. Full search criteria are included in Supplemental Table 1.

### Inclusion and exclusion criteria

Study inclusion criteria included English language RCTs in adults aged 18 y or older who were apparently healthy or who had overweight/obesity, type 2 diabetes mellitus, metabolic syndrome, hyper/dyslipidemia, or hypertension. Trials in participants with other chronic diseases at baseline (e.g., cancer) were excluded. Interventions included fresh, unprocessed, or minimally processed beef compared with a control diet without beef or with a lower amount of beef. Exclusion criteria included cross-sectional, retrospective, and prospective cohort studies or any other observational study design. Studies in children (<18 y), pregnant/lactating females, and animal studies were excluded, as well as any studies examining a mixture of red meats, or where the type of red meat was not specified. Additionally, interventions with only processed beef, beef components in the form of dietary supplements, or beef administered nonorally were also excluded.

### Screening, data extraction, and study quality assessment

Screening of titles and abstracts was conducted by 2 independent reviewers (OMP, LMS). Potentially eligible publications were obtained for full-text review by the same independent reviewers (OMP, LMS). Any questions regarding eligibility were resolved by discussion with the research team. Reference lists from eligible publications and recent systematic reviews on red meat and cardiometabolic health were reviewed to determine any additional studies not identified in the search. Population, intervention, comparator, and outcome (PICO) data were extracted from eligible full-text publications by 2 independent reviewers (OMP, LMS) and crosschecked. Discrepancies were resolved by referring to the original article and discussion within the research team. Data contained in graphs were quantified using Engauge Digitizer software version 4.1 (https://markummitchell.github.io/engauge-digitizer/). The Cochrane Risk of Bias (RoB) tool was used to evaluate RoB for each study using the appropriate versions for parallel or crossover studies [21]. Data extracted included study design; location; sample size; population age, sex, health, and weight status; amount and description of beef consumed; background diet; comparator diet; trial duration; funding source; attrition and reason for withdrawal; and outcomes measured. When available, outcome data for the intention-to-treat population were used to minimize bias due to attrition.

### Statistical analysis

Where sufficient published results were available (≥3 comparisons in RCTs), meta-analyses were completed using Comprehensive Meta-Analysis software version 3 (Biostat). Initially, the intention was to calculate weighted mean differences to retain the use of units in the analyses; however, the use of geometric means ± 1 SD in some of the results precluded our ability to use weighted differences, resulting in the use of standardized mean difference (SMD). The primary analysis used pooled SMD estimates and 95% CIs for blood lipid–related and blood pressure outcomes. Statistical significance for individual studies and pooled SMD was confirmed when the 95% CI did not include the null value of 0 (i.e., *P* < 0.05). Studies were weighted according to the inverse of the variance of each study’s effect using random-effect models. Random-effect models were chosen based on heterogeneity in the study length, intervention and comparator, populations, and study designs. SMDs and corresponding standard errors for individual studies were computed by the software using methods for independent groups and matched groups described by Borenstein et al. [22] with an imputed between-treatment correlation of 0.50 for matched groups. To avoid potential bias from using a single imputed correlation value, sensitivity analyses using correlation values of 0.3 and 0.7 were conducted as recommended by Balk et al. [23]. Neither analysis was markedly different from the main analysis, so only results using a between-treatment correlation of 0.5 are presented. For multiple comparisons within a study that shared a common active or control, individual effect sizes and variances were computed for all comparisons, and a pooled effect size estimate was computed as the weighted average of the individual effect size estimates. The corresponding variances were computed as the mean of 2 or more effect size estimates using between-comparison correlations equivalent to the weighted average of the between-active correlation (beef) and the between control correlation [24]. For studies with multiple comparisons, forest plot representations include each comparison separately, but a single pooled effect size estimate and variance was used in the analysis. Heterogeneity across studies was determined using Cochran *Q* and *I*^2^ statistic. An *I*^2^ value of ≥40% was used to designate moderate or higher heterogeneity as defined by the Cochrane Handbook [25].

Sensitivity analyses included removal of 1 study at a time and removal of weight loss studies. Subgroup analyses included study design (crossover and parallel), weight status (healthy, healthy/overweight, overweight/obese, and mix of all weights), health risk (healthy and ≥1 indicator of impaired cardiometabolic health or type 2 diabetes), sex (male/female), amount of beef consumed (≤median or >median of included studies), length of intervention (≤median or >median of included studies), attrition (<25% or ≥25%), study quality as determined by Cochrane RoB analysis (low and high/some concerns), funding source (beef organizations and nonbeef organizations), comparator diet (plant protein, animal protein, carbohydrate, and mix of proteins), and year of publication (before 2000, 2000–2010, and after 2010). No pooled effect sizes were calculated for subgroups when <3 comparisons were available. The magnitude of each effect size was interpreted as <0.40 = small, 0.40–0.70 = moderate, and >0.70 = large [26]. Publication bias was assessed through visual examination of funnel plots, as well as Egger regression method when there were ≥10 studies.

## Results

**FIGURE 1:**
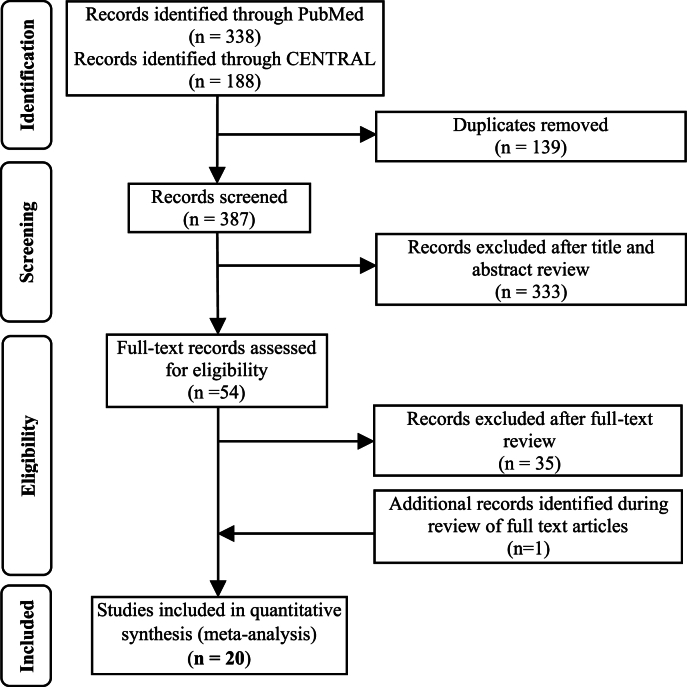
PRISMA flow chart of the study selection process.

The results of the literature search process are shown in Figure 1. Following title and abstract review, 44 articles were determined eligible for full-text review. Thirty-five of these publications were excluded from the meta-analysis, primarily due to inclusion of other red meats (e.g., pork and lamb) or lack of specificity on the type of red meat included in the diet. One additional publication was identified during review of references. Supplemental Table 2 includes the list of ineligible full-text articles and the criteria for their exclusion. Quantitative data were extracted from 20 full-text publications for inclusion in the meta-analysis [[27], [28], [29], [30], [31], [32], [33], [34], [35], [36], [37], [38], [39], [40], [41], [42], [43], [44], [45], [46]].

**FIGURE 2:**
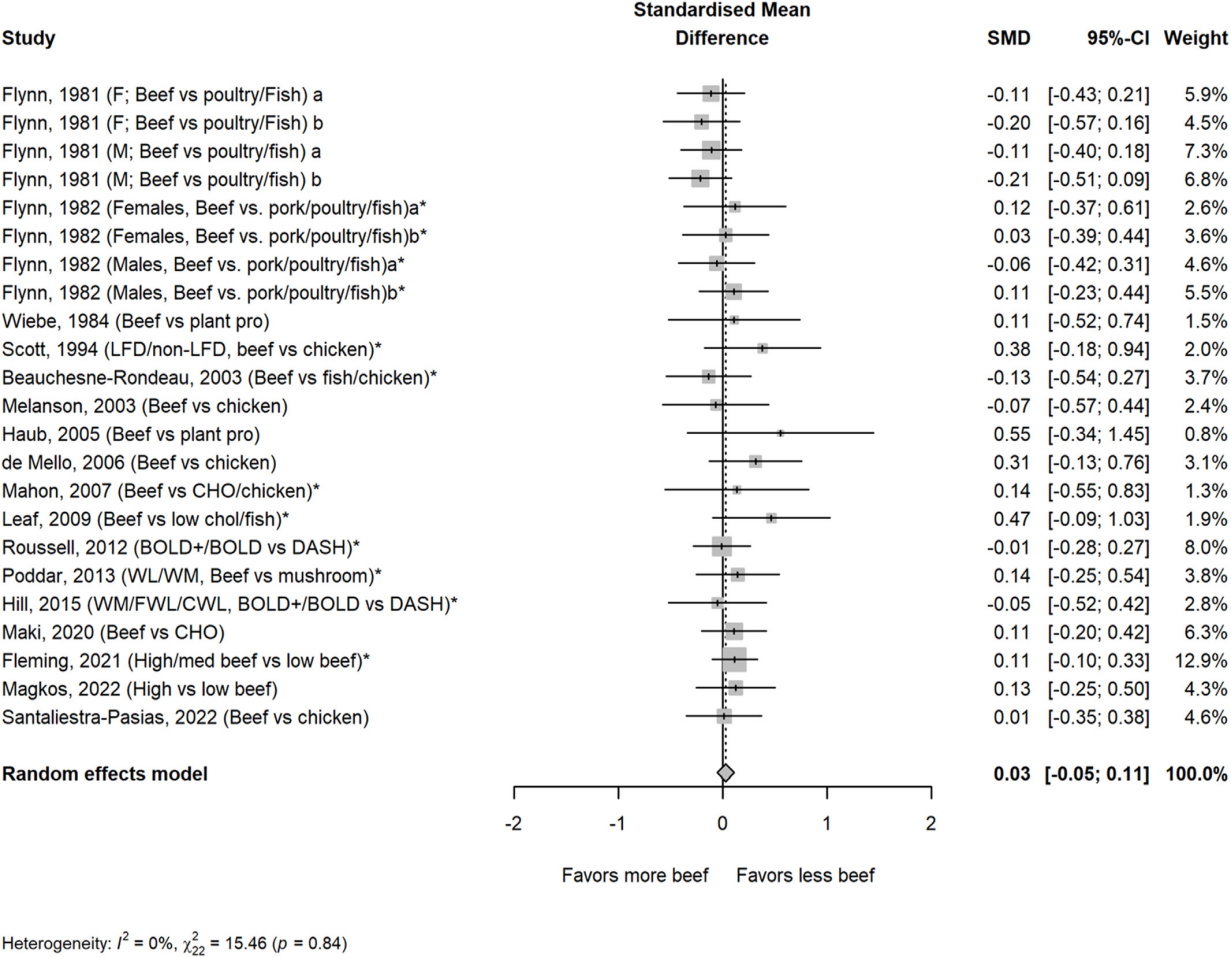
Effect of higher beef intake on total cholesterol. Values are standardized mean differences (SMDs) of total cholesterol between the beef diet and diets with less or no beef. Effect sizes for correlated comparisons within study were pooled prior to running the model. Pooled effect *P* = 0.49. BOLD, Beef in an Optimal Lean Diet; CHO, carbohydrate; CWL, controlled weight loss; DASH, dietary approaches to stop hypertension; FWL, free living weight loss; LFD, low-fat diet; WL, weight loss; WM, weight maintenance.

**FIGURE 3:**
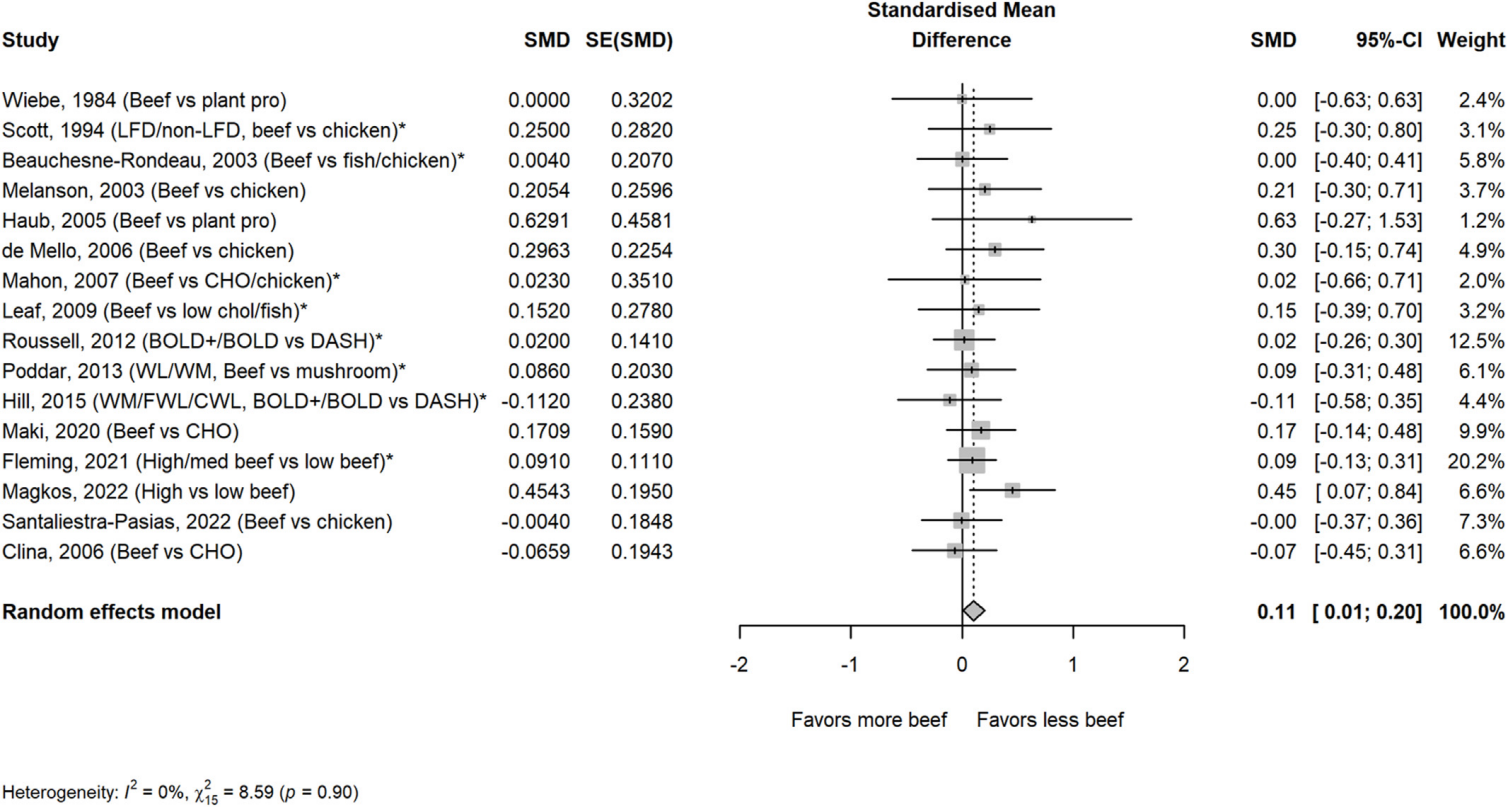
Effect of higher beef intake on LDL-cholesterol. Values are standardized mean differences (SMDs) of LDL-cholesterol between the beef diet and diets with less or no beef. Effect sizes for correlated comparisons within study were pooled prior to running the model. Pooled effect *P* = 0.03. See Supplemental Figure 8 for sensitivity analysis. BOLD, Beef in an Optimal Lean Diet; CHO, carbohydrate; CWL, controlled weight loss; DASH, Dietary Approaches to Stop Hypertension; FWL, free living weight loss; LFD, low-fat diet; WL, weight loss; WM, weight maintenance.

**FIGURE 4:**
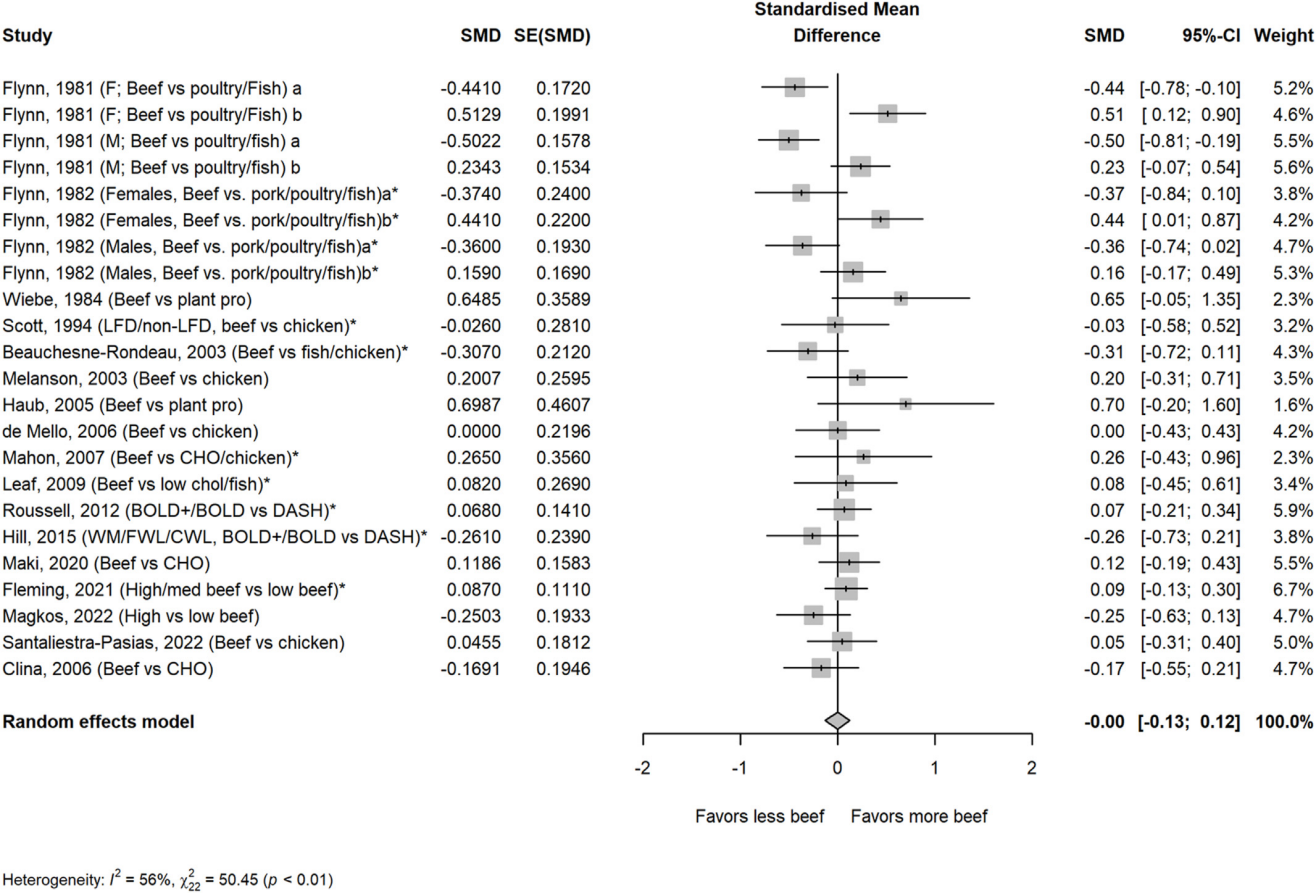
Effect of higher beef intake on HDL-cholesterol. Values are standardized mean differences (SMDs) of HDL-cholesterol between the beef diet and diets with less or no beef. Effect sizes for correlated comparisons within study were pooled prior to running the model. Pooled effect *P* = 0.99. BOLD, Beef in an Optimal Lean Diet; CHO, carbohydrate; CWL, controlled weight loss; DASH, Dietary Approaches to Stop Hypertension; FWL, free living weight loss; LFD, low-fat diet; WL, weight loss; WM, weight maintenance.

**FIGURE 5:**
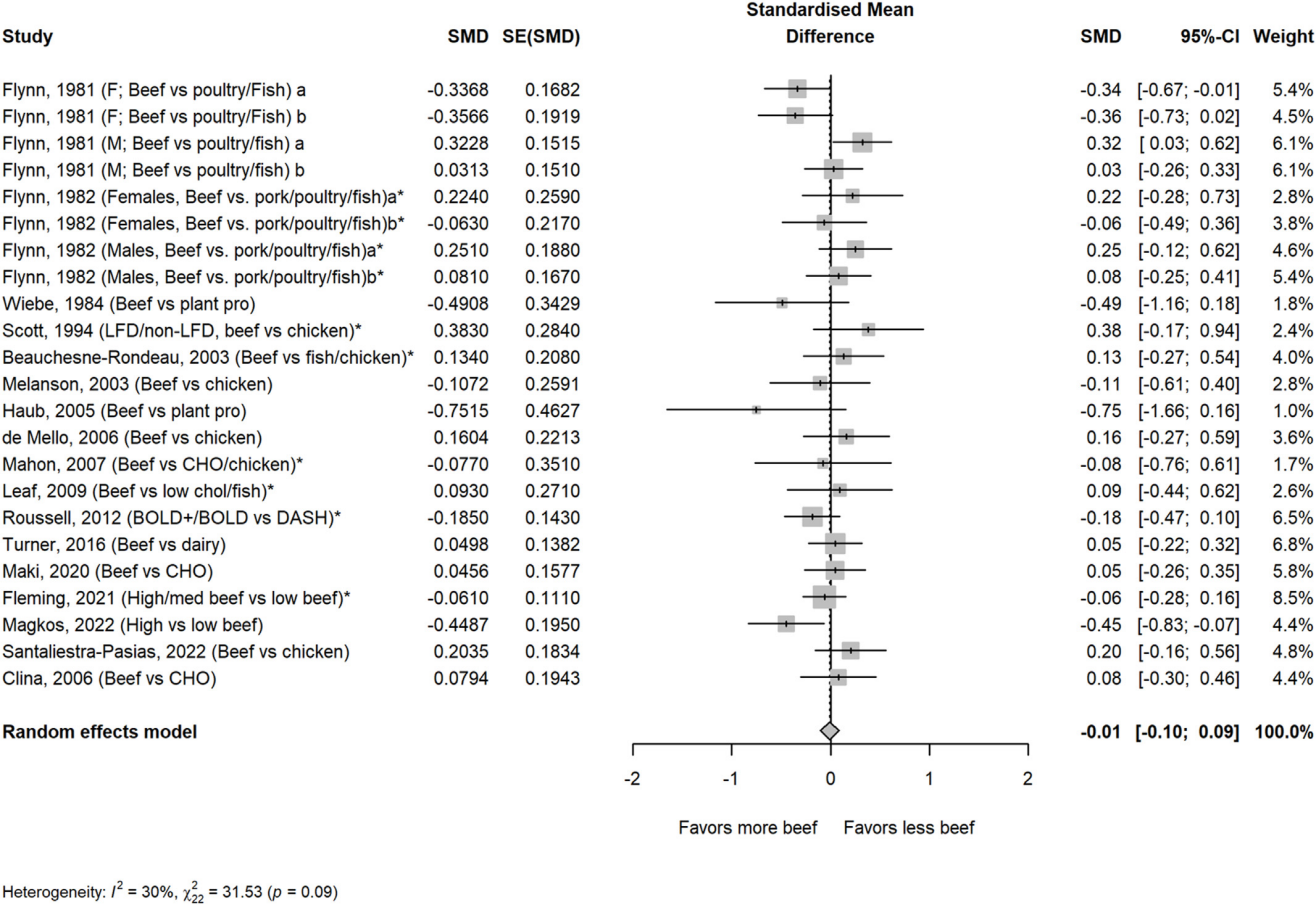
Effect of higher beef intake on triglycerides (TGs). Values are standardized mean differences (SMDs) of TG between the beef diet and diets with less or no beef. Effect sizes for correlated comparisons within study were pooled prior to running the model. Pooled effect *P* = 0.86. BOLD, Beef in an Optimal Lean Diet; CHO, carbohydrate; CWL, controlled weight loss; DASH, Dietary Approaches to Stop Hypertension; FWL, free living weight loss; LFD, low-fat diet; WL, weight loss; WM, weight maintenance.

The average amount of beef in the higher beef treatments was 161 g/d or ∼2 servings/d. Most comparator diets provided 0 g of beef, but comparator diets that allowed for small amounts of beef averaged 24 g/d or <1 serving/d. Beef intake did not impact circulating lipoprotein lipids or lipoproteins assessed, including TC, HDL-cholesterol, TG, non–HDL-cholesterol, apolipoprotein A or B, VLDL-cholesterol, and cholesterol ratios, with the exception of LDL-cholesterol in the beef diet compared with that in comparator diets consisting of less or no beef (FIGURE 1, FIGURE 2, FIGURE 3, FIGURE 4, FIGURE 5 and Supplemental Figures S1–S5). Beef consumption had a small but significant effect on LDL-cholesterol (SMD: 0.11; 95% CI: 0.008, 0.20; *P* = 0.03), indicating modestly higher levels with greater intake of beef (Figure 3). A 1-study-removed sensitivity analysis indicated that the study by Magkos et al. [36] influenced these results because its removal attenuated the effect of dietary beef on LDL-cholesterol (SMD: 0.08; 95% CI: −0.02, 0.18; *P* = 0.11) (Supplemental Figure S6).

Magkos et al. [36] provided a very low–calorie diet (VLCD) of 600–770 kcal/d for an 8-wk weight loss lead in, followed by a 12-wk weight maintenance diet with 25 g beef/d or 150 g beef/d. The reported mean ± SE baseline values for LDL-cholesterol in this study were higher in the group consuming less beef (125.3 ± 4.6 mg/dL) than those in the group consuming more beef (112.9 ± 4.6 mg/dL). By the end of the study, both groups had comparable mean LDL-cholesterol (112.5 ± 3.5 mg/dL compared with 112.9 ± 3.5 mg/dL, respectively) but the reduction was larger in the group consuming less beef due to the higher baseline value (Supplemental Figure S7). This meta-analysis also used the reported baseline LDL-cholesterol values taken prior to the commencement of the VLCD, rather than the LDL-cholesterol values reported after the weight loss phase and just prior to the commencement of the 12-wk, weight maintenance diet containing beef. Thus, a post hoc sensitivity analysis was conducted using the LDL-cholesterol values reported at the end of the VLCD weight loss phase as baseline values. The sensitivity analysis resulted in a reduction in the pooled effect size for LDL-cholesterol (SMD: 0.09; 95% CI: −0.01, 0.19; *P* = 0.08) (Supplemental Figure S8).

One study removed sensitivity analyses for other outcomes showed no significant influence of individual studies on the effect size. Similarly, removal of weight loss studies did not significantly alter the results. Visual inspection of funnel plots and Egger regression showed no evidence of publication bias for any outcomes (Supplemental Figures S9–S12). Results of the Cochrane RoB assessment can be found in Supplemental Table 3.

Subgroup analyses show no significant effects of beef diets compared with comparator diets on any assessed lipid profile parameters with the exception of study quality for LDL-cholesterol and sex for TG (Table 1). Studies with low RoB showed a small but significant (*P* = 0.03) effect for less beef to be associated with lower LDL-cholesterol. This effect may be the result of the study of Magkos et al. [36] being categorized as a low RoB study, and when a post hoc subgroup analysis was conducted using the alternate, post VLCD, 8-wk weight loss LDL-cholesterol baseline, the impact of study quality on LDL-cholesterol outcomes was no longer significant (Supplemental Figure S13). For females, but not males, TG levels were lower with greater beef intake (SMD: −0.19; 95% CI: −0.36, −0.01; *P* = 0.04) (Table 1).

**TABLE 1:** Subgroup analysis for effect of beef, compared with less or no beef intake, on total cholesterol, LDL-cholesterol, HDL-cholesterol, and triglycerides.

Outcome and subgroups	Effect estimate		*I*^2^ (%)	*P*
	SMD^1^	95% CI		
*Total cholesterol*				
Study design				
Crossover	0.01	−0.08, 0.09	0	0.86
Parallel	0.12	−0.06, 0.31	0	0.20
Between-subgroup heterogeneity (*Q* = 1.2, *P* = 0.271)				
Body weight status				
Healthy	−0.12	−0.26, 0.02	0	0.10
Healthy/overweight	0.08	−0.08, 0.23	0	0.32
Overweight/obese	0.08	−0.09, 0.25	0	0.36
Mixed weights	0.12	−0.05, 0.29	6	0.17
Between-subgroup heterogeneity (*Q* = 5.9, *P* = 0.116)				
Health risk^2^				
Healthy	0.00	−0.09, 0.09	0	0.97
Metabolic dysfunction	0.09	−0.05, 0.24	0	0.21
Between-subgroup heterogeneity (*Q* = 1.1, *P* = 0.298)				
Amount of beef (median split)				
>142 g	0.01	−0.08, 0.10	0	0.33
≤142 g	0.09	−0.09, 0.27	0	0.76
Between-subgroup heterogeneity (*Q* = 0.6, *P* = 0.458)				
Sex				
Male	−0.03	−0.17, 0.10	0	0.64
Female	−0.06	−0.23, 0.12	0	0.52
Between-subgroup heterogeneity (*Q* = 0.1, *P* = 0.829)				
Comparator diet				
Animal protein	−0.01	−0.10, 0.07	0	0.81
Plant protein	0.19	−0.13, 0.50	0	0.25
Carbohydrate	0.14	−0.09, 0.37	0	0.24
Between-subgroup heterogeneity (*Q* = 4.2, *P* = 0.246)				
Study duration (median split)				
>63 d	−0.05	−0.16, 0.06	0	0.37
≤63 d	0.10	−0.02, 0.21	0	0.09
Between-subgroup heterogeneity (*Q* = 3.3, *P* = 0.070)				
Date of study				
Prior to 2000	−0.05	−0.17, 0.07	0	0.41
2000–2010	0.14	−0.08, 0.36	0	0.21
After 2010	0.07	−0.05, 0.19	0	0.23
Between-subgroup heterogeneity (*Q* = 3.2, *P* = 0.199)				
Attrition				
<25%	0.007	−0.08, 0.09	0	0.88
≥25%	0.10	−0.09, 0.29	0	0.32
Between-subgroup heterogeneity (*Q* = 0.7, *P* = 0.400)				
Study quality				
Low RoB	0.08	−0.04, 0.19	0	0.19
Some concerns or high RoB	−0.02	−0.12, 0.09	0	0.78
Between-subgroup heterogeneity (*Q* = 1.3, *P* = 0.249)				
Funding source				
Beef industry	0.00	−0.08, 0.09	0	0.98
Nonbeef source	0.13	−0.08, 0.35	0	0.22
Between-subgroup heterogeneity (*Q* = 1.3, *P* = 0.263)				
*LDL-cholesterol*				
Study design				
Crossover	00.09	−0.03; 0.21	0	0.16
Parallel	0.15	−0.02, 0.31	0	0.09
Between-subgroup heterogeneity (*Q* = 0.3, *P* = 0.573)				
Body weight status				
Healthy	−0.00	−0.32, 0.31	0	0.98
Healthy/overweight	0.16	−0.10, 0.43	0	0.22
Overweight/obese	0.13	−0.03, 0.29	0	0.11
Mixed weights	0.09	−0.07, 0.25	0	0.27
Between-subgroup heterogeneity (*Q* = 0.8, *P* = 0.857)				
Health risk^2^				
Healthy	0.14	−0.00, 0.28	0	0.051
Metabolic dysfunction	0.07	−0.06, 0.21	0	0.29
Between-subgroup heterogeneity (*Q* = 0.4, *P* = 0.520)				
Amount of beef (median split)				
>142 g	0.08	−0.06, 0.22	0	0.28
≤142 g	0.10	−0.03, 0.22	0	0.12
Between-subgroup heterogeneity (*Q* = 0.1, *P* = 0.810)				
Sex				
Male	0.12	−0.15, 0.40	0	0.38
Female	0.14	−0.27, 0.55	0	0.50
Between-subgroup heterogeneity (*Q* = 0.0, *P* = 0.945)				
Comparator diet				
Animal protein	0.06	−0.06, 0.18	0	0.32
Plant protein	0.13	−0.18, 0.45	0	0.41
Carbohydrate	0.17	−0.05, 0.40	18	0.12
Between-subgroup heterogeneity (*Q* = 1.0, *P* = 0.811)				
Study duration (median split)				
≤35 d	0.11	−0.01, 0.23	0	0.08
>35 d	0.10	−0.10, 0.31	23	0.33
Between-subgroup heterogeneity (*Q* = 0.0, *P* = 0.963)				
Date of study				
Prior to 2000	0.14	−0.27, 0.56	0	0.51
2000–2010	0.17	−0.05, 0.38	0	0.12
After 2010	0.09	−0.03, 0.20	0	0.14
Between-subgroup heterogeneity (*Q* = 0.48, *P* = 0.788)				
Attrition				
<25%	0.07	−0.05, 0.19	0	0.28
≥25%	0.17	−0.03, 0.34	0	0.054
Between-subgroup heterogeneity (*Q* = 0.9, *P* = 0.348)				
Study quality				
Low RoB	0.13	0.01, 0.24	0	*0.03*
Some concerns or high RoB	0.05	−0.14, 0.24	0	0.60
Between-subgroup heterogeneity (*Q* = 0.4, *P* = 0.505)				
Funding source				
Beef industry	0.11	−0.00, 0.22	0	0.06
Nonbeef source	0.09	−0.12, 0.31	0	0.39
Between-subgroup heterogeneity (*Q* = 0.01, *P* = 0.912)				
*HDL-cholesterol*				
Study design				
Crossover	0.01	−0.14, 0.16	65	0.91
Parallel	−0.06	−0.26, 0.14	8	0.54
Between-subgroup heterogeneity (*Q* = 0.3, *P* = 0.578)				
Body weight status				
Healthy	0.04	−0.32, 0.41	83	0.81
Healthy/overweight	−0.06	−0.29, 0.17	50	0.62
Overweight/obese	−0.04	−0.22, 0.13	1	0.61
Mixed weights	0.10	−0.06, 0.26	0	0.22
Between-subgroup heterogeneity (*Q* = 2.0, *P* = 0.583)				
Health risk^2^				
Healthy	0.04	−0.15, 0.22	69	0.68
Metabolic dysfunction	−0.03	−0.17, 0.10	0	0.63
Between-subgroup heterogeneity (*Q* = 0.4, *P* = 0.534)				
Amount of beef (median split)				
>142 g	0.02	−0.26, 0.30	48	0.88
≤142 g	−0.04	−0.19, 0.12	62	0.65
Between-subgroup heterogeneity (*Q* = 0.1, *P* = 0.725)				
Sex				
Male	−0.01	−0.29, 0.26	70	0.93
Female	0.09	−0.28, 0.47	75	0.63
Between-subgroup heterogeneity (*Q* = 0.2, *P* = 0.657)				
Comparator diet				
Animal protein	−0.03	−0.17, 0.11	58	0.65
Plant protein	<3 comparisons			
Carbohydrate	0.01	−0.29, 0.31	52	0.97
Between-subgroup heterogeneity (*Q* = 6.6, *P* = 0.087)				
Study duration (median split)				
≤63 d	0.04	−0.07, 0.16	0	0.44
>63 d	−0.04	−0.25, 0.17	71	0.72
Between-subgroup heterogeneity (*Q* = 0.5, *P* = 0.499)				
Attrition				
<25%	−0.01	−0.17, 0.14	63	0.86
≥25%	−0.03	−0.24, 0.17	14	0.75
Between-subgroup heterogeneity (*Q* = 0.02, *P* = 0.880)				
Date of study				
Prior to 2000	0.01	−0.25, 0.27	77	0.96
2000–2010	0.04	−0.19, 0.27	10	0.72
After 2010	0.00	−0.12, 0.12	0	0.96
Between-subgroup heterogeneity (*Q* = 0.1, *P* = 0.959)				
Study quality				
Low RoB	0.03	−0.10, 0.17	21	0.63
Some concerns or high RoB	−0.04	−0.24, 0.17	70	0.73
Between-subgroup heterogeneity (*Q* = 0.3, *P* = 0.575)				
Funding source				
Beef industry	−0.02	−0.16, 0.12	61	0.75
Nonbeef source	0.13	−0.18, 0.43	24	0.41
Between-subgroup heterogeneity (*Q* = 0.8, *P* = 0.378)				
*Triglycerides*				
Study design				
Crossover	0.01	−0.09, 0.11	25	0.81
Parallel	−0.11	−0.40, 0.17	44	0.44
Between-subgroup heterogeneity (*Q* = 0.7, *P* = 0.418)				
Body weight status				
Healthy	−0.06	−0.32, 0.19	67	0.63
Healthy/overweight	0.15	−0.01, 0.30	0	0.67
Overweight/obese	−0.05	−0.21, 0.11	10	0.56
Mixed weights	−0.11	−0.27, 0.05	0	0.18
Between-subgroup heterogeneity (*Q* = 5.7, *P* = 0.129)				
Health risk^2^				
Healthy	−0.04	−0.17, 0.08	43	0.52
Metabolic dysfunction	0.04	−0.10, 0.18	0	0.59
Between-subgroup heterogeneity (*Q* = 0.7, *P* = 0.409)				
Amount of beef (median split)				
>142 g	0.04	−0.23, 0.31	36	0.77
≤142 g	−0.02	−0.13, 0.10	37	0.78
Between-subgroup heterogeneity (*Q* = 0.1, *P* = 0.703)				
Sex				
Male	0.11	−0.06, 0.29	32	0.22
Female	−0.19	−0.36, −0.01	0	*0.04*
Between-subgroup heterogeneity (*Q* = 5.4, ***P* = 0.021**)				
Comparator diet				
Animal protein	0.03	−0.06, 0.13	17	0.51
Plant protein	< 3 comparisons			
Carbohydrate	−0.11	−0.37, 0.16	39	0.43
Between-subgroup heterogeneity (*Q* = 6.4, *P* = 0.095)				
Study duration (median split)				
≤63 d	0.01	−0.09, 0.12	0	0.82
>63 d	−0.05	−0.21, 0.12	52	0.57
Between-subgroup heterogeneity (*Q* = 0.4, *P* = 0.550)				
Attrition				
<25%	0.02	−0.08, 0.13	30	0.69
≥25%	−0.10	−0.34, 0.15	39	0.44
Between-subgroup heterogeneity (*Q* = 0.75, *P* = 0.385)				
Date of study				
Prior to 2000	0.02	−0.16, 0.19	51	0.87
2000–2010	0.02	−0.19, 0.24	0	0.84
After 2010	−0.04	−0.18, 0.09	28	0.53
Between-subgroup heterogeneity (*Q* = 0.4, *P* = 0.822)				
Study quality				
Low RoB	−0.10	−0.24, 0.03	14	0.12
Some concerns or high RoB	0.06	−0.06, 0.18	29	0.34
Between-subgroup heterogeneity (*Q* = 3.2, *P* = 0.075)				
Funding source				
Beef industry	−0.03	−0.14, 0.09	37	0.64
Nonbeef source	0.07	−0.14, 0.27	12	0.52
Between-subgroup heterogeneity (*Q* = 0.6, *P* = 0.430)				

Abbreviations: RoB, risk of bias; SMD, standardized mean difference.

Italic value indicates statistically significant findings.

1 Effect estimates and *P* values from random-effect models.

2 Metabolic dysfunction = participants recruited had ≥1 cardiometabolic risk factor (e.g., hyperlipidemia, hypertension) or had conditions of metabolic syndrome, prediabetes, or type 2 diabetes.

**FIGURE 6:**
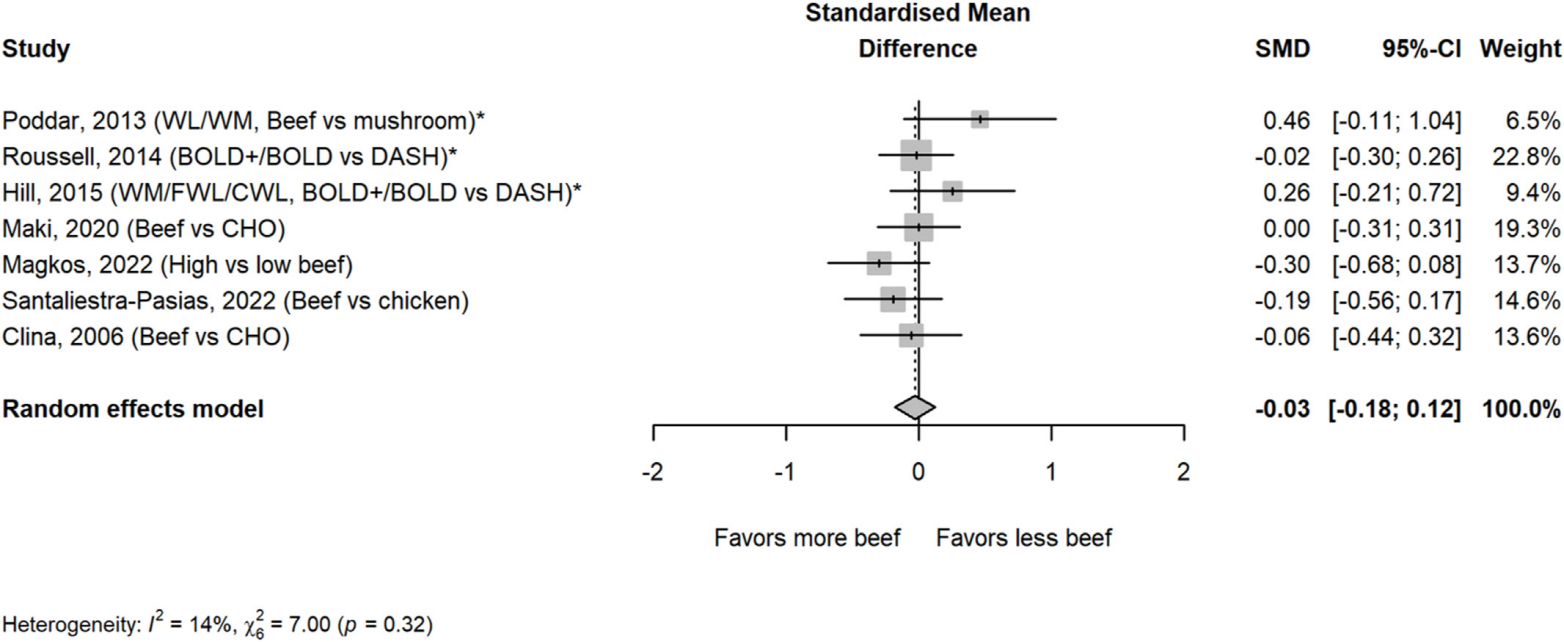
Effect of higher beef intake on systolic blood pressure. Values are standardized mean differences (SMDs) of systolic blood pressure between the beef diet and diets with less or no beef. ∗Effect sizes for correlated comparisons within study were pooled prior to running the model. Pooled effect *P* = 0.73. BOLD, Beef in an Optimal Lean Diet; CHO, carbohydrate; CWL, controlled weight loss; DASH, Dietary Approaches to Stop Hypertension; FWL, free living weight loss; WL, weight loss; WM, weight maintenance.

**FIGURE 7:**
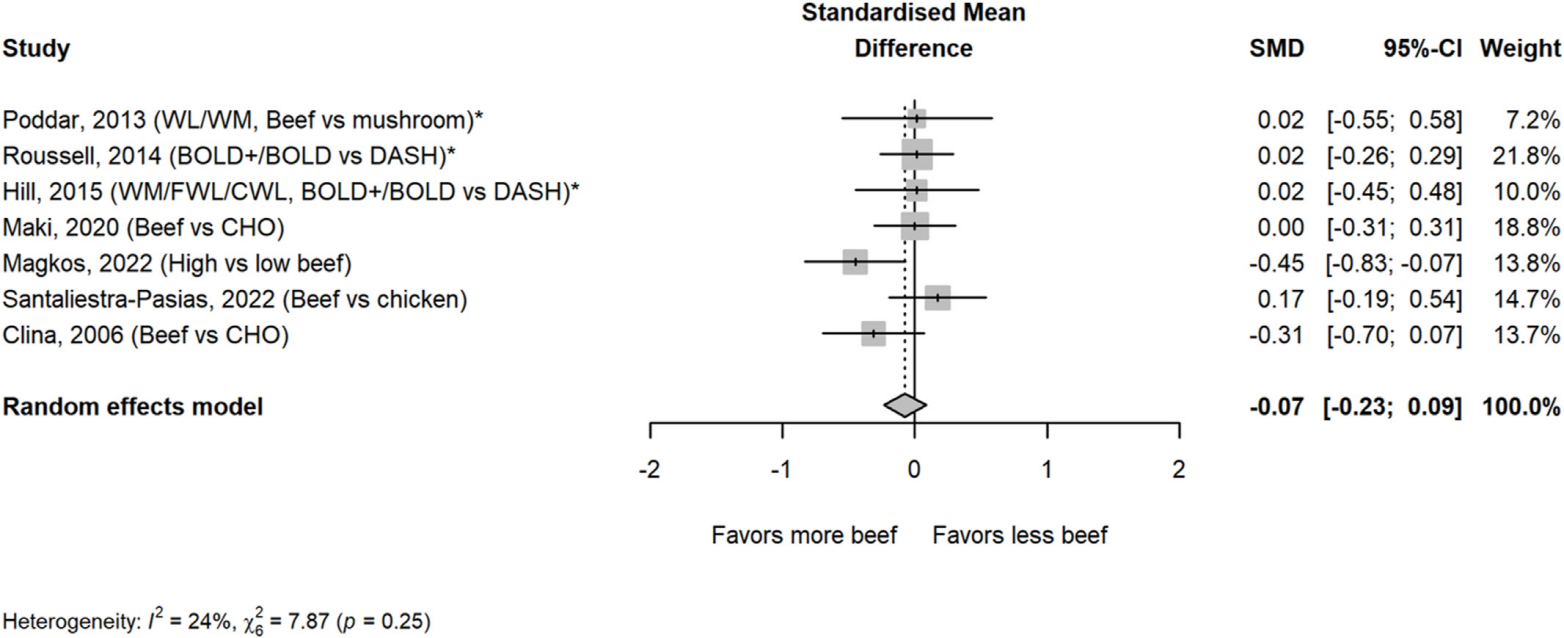
Effect of higher beef intake on diastolic blood pressure. Values are standardized mean differences (SMDs) of diastolic blood pressure between the beef diet and diets with less or no beef. ∗Effect sizes for correlated comparisons within study were pooled prior to running the model. Pooled effect *P* = 0.38. BOLD, Beef in an Optimal Lean Diet; CHO, carbohydrate; CWL, controlled weight loss; DASH, Dietary Approaches to Stop Hypertension; FWL, free living weight loss; WL, weight loss; WM, weight maintenance.

Beef intake had no significant impact on blood pressure measures, including systolic (Figure 6) and diastolic blood pressures (Figure 7). Sensitivity analyses of removal of 1 study at a time and removal of weight loss studies also revealed no significant effects. No significance was found for any blood pressure measure in any of the subgroups analyzed (Table 2).

**TABLE 2:** Subgroup analysis for effect of beef, compared with less or no beef intake, on systolic and diastolic blood pressure values.

Outcome and subgroups	Effect estimate		*I*^2^ (%)	*P*
	SMD^1^	95% CI		
*Systolic blood pressure*				
Study design				
Crossover	−0.05	−0.23, 0.13	0	0.56
Parallel	0.04	−0.27, 0.36	51	0.79
Between-subgroup heterogeneity (*Q* = 0.3, *P* = 0.597)				
Body weight status				
Healthy	<3 comparisons			
Healthy/overweight	<3 comparisons			
Overweight/obese	0.02	−0.20, 0.24	34	0.86
Mixed weights	<3 comparisons			
Between-subgroup heterogeneity (*Q* = 1.0, *P* = 0.622)				
Health risk^2^				
Healthy	−0.07	−0.46, 0.32	60	0.74
Metabolic dysfunction	0.02	−0.15, 0.18	0	0.86
Between-subgroup heterogeneity (*Q* = 0.1, *P* = 0.707)				
Amount of beef (median split)				
>127 g	0.06	−0.31, 0.42	44	0.76
≤127 g	−0.02	−0.27, 0.23	45	0.89
Between-subgroup heterogeneity (*Q* = 0.1, *P* = 0.741)				
Comparator diet				
Animal protein	−0.02	−0.23, 0.19	8	0.86
Plant protein	<3 comparisons			
Carbohydrate	−0.10	−0.30, 0.10	0	0.33
Between-subgroup heterogeneity (*Q* = 3.3, *P* = 0.192)				
Study duration (median split)				
≤42 d	0.02	−0.17, 0.21	0	0.85
>42 d	−0.09	−0.34, 0.15	31	0.45
Between-subgroup heterogeneity (*Q* = 0.5, *P* = 0.478)				
Attrition				
<25%	−0.02	−0.23, 0.19	8	0.86
≥25%	−0.03	−0.27, 0.22	38	0.84
Between-subgroup heterogeneity (*Q* = 0.00, *P* = 0.966)				
Study quality				
Low RoB	−0.03	−0.22, 0.15	12	0.73
Some concerns or high RoB	0.01	−0.32, 0.34	44	0.96
Between-subgroup heterogeneity (*Q* = 0.1, *P* = 0.827)				
Funding source				
Beef industry	−0.04	−0.19, 0.12	0	0.64
Nonbeef source	<3 comparisons			
Between-subgroup heterogeneity (*Q* = 0.2, *P* = 0.692)				
*Diastolic blood pressure*				
Study design				
Crossover	0.05	−0.13, 0.23	0	0.59
Parallel	−0.23	−0.46, −0.01	8	*0.04*
Between-subgroup heterogeneity (*Q* = 3.7, *P* = 0.05)				
Body weight status				
Healthy	<3 comparisons			
Healthy/overweight	<3 comparisons			
Overweight/obese	−0.16	−0.36, 0.04	17	0.11
Mixed weights	<3 comparisons			
Between-subgroup heterogeneity (*Q* = 2.9, *P* = 0.237)				
Health risk^2^				
Healthy	−0.09	−0.51, 0.32	64	0.65
Metabolic dysfunction	−0.05	−0.22, 0.12	0	0.54
Between-subgroup heterogeneity (*Q* = 0.0, *P* = 0.855)				
Amount of beef (median split)				
>127 g	0.10	−0.16, 0.36	0	0.45
≤127 g	−0.18	−0.42, 0.06	38	0.14
Between-subgroup heterogeneity (*Q* = 2.4, *P* = 0.122)				
Comparator diet				
Animal protein	0.06	−0.14, 0.26	0	0.53
Plant protein	<3 comparisons			
Carbohydrate	−0.23	−0.51, 0.04	44	0.09
Between-subgroup heterogeneity (*Q* = 3.0, *P* = 0.224)				
Study duration (median split)				
≤42 d	−0.00	−0.19, 0.19	0	0.97
>42 d	−0.12	−0.45, 0.21	60	0.48
Between-subgroup heterogeneity (*Q* = 0.4, *P* = 0.549)				
Attrition				
<25%	0.06	−0.14, 0.26	0	0.53
≥25%	−0.20	−0.43, 0.04	28	0.10
Between-subgroup heterogeneity (*Q* = 2.77, *P* = 0.096)				
Study quality				
Low RoB	−0.09	−0.30, 0.12	33	0.41
Some concerns or high RoB	−0.05	−0.36, 0.27	40	0.78
Between-subgroup heterogeneity (*Q* = 0.1, *P* = 0.818)				
Funding source				
Beef industry	−0.13	−0.32, 0.06	30	0.18
Nonbeef source	<3 comparisons			
Between-subgroup heterogeneity (*Q* = 2.0, *P* = 0.162)				

Abbreviations: RoB, risk of bias; SMD, standardized mean difference.

Italic value indicates statistically significant findings.

1 Effect estimates and *P* values from random-effect models.

2 Metabolic dysfunction = participants recruited had ≥1 cardiometabolic risk factor (e.g., hyperlipidemia, hypertension) or had conditions of metabolic syndrome, prediabetes, or type 2 diabetes.

## Discussion

This systematic review and meta-analysis of RCTs evaluating the effects of beef intake on CVD risk factors found no effect of beef intake on circulating lipids, apolipoproteins, and blood pressures, with the exception of a small effect on LDL-cholesterol levels favoring lower dietary beef intake. The effect size of 0.11 corresponds to ∼2.7 mg/dL difference between diets with more and those with less beef. This effect is partially attributable to 1 study, as removal of this study attenuated the effect on LDL-cholesterol [36].

Although a single study may have partially influenced the significant effect on LDL-cholesterol, it is also plausible that beef intake may mildly affect LDL-cholesterol levels due to its dietary cholesterol content. It is less likely that saturated fat from beef is an important driver of an increase in LDL-cholesterol because the fatty acid profile of unprocessed beef includes more cholesterol-lowering or neutral fatty acids than cholesterol-raising fatty acids [47,48]. According to USDA Food Data Central, a serving of 80% lean ground beef has almost twice the content of cholesterol-lowering fatty acids (monosaturated and polyunsaturated fatty acids; 9.4 g/serving) as cholesterol-raising saturated fatty acids (12:0 + 14:0 + 16:0; 4.9 g/serving) [49]. More than half of the studies included in the meta-analysis also attempted to match saturated fat content between the test and comparator diet. Animal-based foods, including beef, are also a source of dietary cholesterol, and a meta-regression completed by Vincent et al. [50] demonstrated an increase of 3.27 mg/dL in LDL-cholesterol for each 100-mg/d increase in dietary cholesterol (linear model for intake ≤400 mg/d). A serving of beef contains 75–85 mg dietary cholesterol, which would be expected to raise LDL-cholesterol by 2.5 to 2.8 mg/dL compared with a cholesterol-free comparator. In these studies, the average difference in dietary cholesterol between beef-based and plant-based diets was 82 mg/d, approximating a serving of beef. Studies have also shown lean beef intake to shift LDL-cholesterol toward larger, more buoyant LDL particles, which may explain the observed increase in LDL-cholesterol compared with no significant effect on ApoB (pooled SMD: 0.05; 95% CI: −0.08, 0.18), the main structural protein in LDL particles [38,[51], [52], [53]].

The results presented in this study are generally consistent with those from previous meta-analyses assessing the effects of beef or red meat intake on blood lipids [7,10,11,54,55]. Specifically, a dose–response meta-analysis found no significant effects of red meat intake on blood lipids or apolipoproteins for intake levels ≤500 g/d (17.6 oz/d) compared with comparator control diets, although they concluded that substituting red meat with high-quality plant protein sources can reduce LDL-cholesterol by ∼7.7 mg/dL [11]. The authors of a 2017 systematic meta-analysis of 24 RCTs assessing the effect of ≥0.5 servings/d of red meat, compared with <0.5 servings/d, on blood lipoprotein lipids and blood pressures also reported no effects [55]. A meta-analysis of 20 RCTs reported that compared with white meat or whole grain-based diets, red meat diets modestly increased LDL-cholesterol (∼4.4 mg/dL), but this did not reach significance [10]. Specific to beef, a previous analysis indicated that beef intake has a similar effect on lipoprotein lipids as fish and/or poultry intake [54].

Although the evidence from RCTs and meta-analyses have not consistently reported a causal relationship of red meat intake and increased blood lipids, observational studies have reported positive associations of red meat intake and adverse CVD outcomes including CVD mortality and risk of total and ischemic stroke [[3], [4], [5]]. The difference in these findings could be due to residual confounding in such studies. For example, people that regularly consume red meat also tend to be more inactive and eat fewer fruits, vegetables and whole grains, which may contribute to increased risk of CVD risk or mortality [3,56]. Although intake of other foods or physical activity is often adjusted for in cohort studies, the measurement tools used for assessment are often imprecise. Alternatively, there could be mechanisms other than changes in traditional cardiometabolic disease risk factors measured in this meta-analysis that mediate an adverse effect of red meat intake on cardiometabolic outcomes, such as increased levels of trimethylamine oxide [57] or heme iron [58]. Finally, some observational studies include processed and unprocessed red meat exposure together when assessing the relationship to CVD risk [6,7], whereas this meta-analysis included only RCTs with lean, unprocessed beef.

Bias can influence the findings of meta-analyses that pool results from clinical trials; therefore, we evaluated several sources of potential bias, including study quality, attrition, publication bias, and funding source. Study quality, as determined by the Cochrane RoB tool, did not have a substantial impact on the results, with the exception of LDL-cholesterol, which is likely attributable to 1 influential study with low RoB as discussed previously [36]. Similarly, attrition rates did not impact the results for any outcomes, although 5 studies (predominantly weight loss studies) reported attrition rates ≥25%. One study [36], reported a greater proportion of individuals withdrew from the intervention with less beef (25 g/d) than those from that with more beef (125 g/d), suggesting possible challenges in the feasibility of reducing red meat intake over the long term in the diets of individuals from industrialized countries. No publication bias was detected based on funnel plots and Egger regression, and subgroup analyses revealed no bias based on funding source. In fact, 71% of studies funded by the beef industry had low RoB compared with 40% of studies not funded by the beef industry.

The inclusion of multiple subgroup and sensitivity analyses strengthen this report, as does the assessment of multiple sources of potential bias. Including only beef is both a strength of this study because beef accounts for the largest fraction of red meat intake in the United States, and a limitation because the results cannot be extrapolated to other meat sources. These results are also limited to unprocessed beef consumption and do not include processed beef, which may be contain additional ingredients that could influence CVD risk, such as nitrates, nitrites, and sodium. In fact, several meta-analyses of cohort studies consistently report positive associations of processed meat intake and CVD-related mortality, but the associations are less consistent with unprocessed red meat [59]. Another limitation is that this meta-analysis examined only blood lipids, apolipoproteins, and blood pressures; other cardiometabolic risk factors, such as inflammatory markers, functional markers, measures of subclinical atherosclerosis, or insulin resistance were not assessed [60]. There were very few studies assessing outcomes such as non–HDL-cholesterol or cholesterol ratios, which are determined by calculation and should be easy to include in studies. Most research suggests that non–HDL-cholesterol is a better predictor on coronary atherosclerosis than LDL-cholesterol, and future studies should consider including this outcome [61].

In summary, the results of this analysis showed no meaningful effect of daily unprocessed beef intake, compared with diets with less or no beef, on circulating lipoprotein lipids, apolipoproteins, and blood pressures, except for a small effect to increase the LDL-cholesterol concentration by ∼2.7 mg/dL. Given that unprocessed beef has minimal to no impact on these CVD risk factors but is a significant source of highly bioavailable protein as well as iron, zinc, and selenium, its inclusion in the diet may help improve dietary nutrient profiles without significantly affecting lipids or blood pressures. Future studies and meta-analyses should examine how beef affects other cardiometabolic disease risk factors, including insulin resistance, glucose intolerance, and inflammatory markers, to provide clearer guidelines on beef consumption and cardiometabolic health.

## Author contributions

The authors’ responsibilities were as follows – KCM, LMS: designed the research; LMS, OP: conducted the research; MLW: analyzed data; LMS, OMP: wrote the article; KCM: had primary responsibility for the final content; and all authors: read and approved the final manuscript.

## Funding

This study was supported by the Beef Checkoff. The funding sponsor provided comments on early aspects of the study design. A report was shared with the sponsor prior to submission. The final decision for all aspects of the study and the manuscript content were those of the authors alone.

## Data availability

Data described in the manuscript, code book, and analytic code will be made available upon reasonable request to the corresponding author.

## Conflict of interest

KCM reports financial support was provided by Beef Checkoff and a relationship with Beef Checkoff that includes funding grants. The other authors report no conflicts of interest.
