# Supplementary material for: Beef Consumption and Cardiovascular Disease Risk Factors: A Systematic Review and Meta-analysis of Randomized Controlled Trials
Source: Curr Dev Nutr. 2024 Nov 2;8(12):104500. doi: 10.1016/j.cdnut.2024.104500 (PMC11621491; doi:10.1016/j.cdnut.2024.104500)
Supplement: multimedia component 1 [file mmc1.docx]

**Beef Consumption and Cardiovascular Risk Factors: A Systematic Review and Meta-Analysis of Randomized Controlled Trials**

**LM Sanders, et al.**

**Supplemental Figures and Tables**

**Supplemental Table 1**. Full search criteria used for PubMed and CENTRAL database searches.

| **Terms used to identify studies** | **Filters** |
| --- | --- |
| **Key search terms for beef**  “Beef” OR “red meat” OR “meat proteins” OR “meat products” OR “fresh meat” OR “unprocessed meat” OR “minimally processed meat”  **Key search terms for cardiometabolic health**  “cardiovascular” OR “hypertension” OR “blood pressure” OR “hyperlipidemia” OR “dyslipidemia” OR “cholesterol” OR “blood lipids” OR “hypertriglyceridemia” OR “triglycerides” OR “apolipoprotein” OR “lipoprotein”  **Key search terms for trials**  randomized controlled trial* OR controlled clinical trial* OR RCT OR random OR placebo* | English |

**Supplemental Table 2.** Ineligible articles after full text review and their reason for exclusion.

| **Title** | **Authors** | **reason for excluding** |
| --- | --- | --- |
| Differences in postprandial inflammatory responses to a 'modern' v. traditional meat meal: a preliminary study. | Arya F, Egger S, Colquhoun D, Sullivan D, Pal S, Egger G. | No measure of variance provided for the outcome variable (triacylglycerol). |
| Soy inclusion in the diet improves features of the metabolic syndrome: a randomized crossover study in postmenopausal women. | Azadbakht L, Kimiagar M, Mehrabi Y, Esmaillzadeh A, Padyab M, Hu FB, Willett WC. | “Red meat” was not clearly defined such that beef may not have been the exclusive source of “red meat.” |
| Effects of red meat, white meat, and nonmeat protein sources on atherogenic lipoprotein measures in the context of low compared with high saturated fat intake: a randomized controlled trial. | Bergeron N, Chiu S, Williams PT, S MK, Krauss RM. | “Red meat” included pork as a source; beef was not exclusive source. |
| Protein-enriched diet, with the use of lean red meat, combined with progressive resistance training enhances lean tissue mass and muscle strength and reduces circulating IL-6 concentrations in elderly women: a cluster randomized controlled trial. | Daly RM, O'Connell SL, Mundell NL, Grimes CA, Dunstan DW, Nowson CA. | “Red meat” consisted of veal, lamb and beef; beef was not exclusive source. |
| Comparison of the effects of lean red meat vs lean white meat on serum lipid levels among free-living persons with hypercholesterolemia: a long-term, randomized clinical trial. | Davidson MH, Hunninghake D, Maki KC, Kwiterovich PO, Jr., Kafonek S. | “Red meat” consisted of veal, pork, and beef; beef was not exclusive source. |
| The effects of substituting red and processed meat for mycoprotein on biomarkers of cardiovascular risk in healthy volunteers: an analysis of secondary endpoints from Mycomeat. | Farsi DN, Gallegos JL, Finnigan TJA, Cheung W, Munoz JM, Commane DM. | Processed meat included in the “red meat” test diet. |
| Effect of lean red meat combined with a multicomponent exercise program on muscle and cognitive function in older adults: a 6-month randomized controlled trial. | Formica MB, Gianoudis J, Nowson CA, O'Connell SL, Milte C, Ellis KA, Daly RM. | “Red meat” consisted of veal, lamb, and beef; beef was not exclusive source. |
| Effect of protein ingestion on the glucose appearance rate in people with type 2 diabetes. | Gannon MC, Nuttall JA, Damberg G, Gupta V, Nuttall FQ. | Dose response study that used water as control; not a dietary intervention study. |
| Effect of a chicken-based diet on renal function and lipid profile in patients with type 2 diabetes: a randomized crossover trial. | Gross JL, Zelmanovitz T, Moulin CC, De Mello V, Perassolo M, Leitão C, Hoefel A, Paggi A, Azevedo MJ. | The study population included both normoalbuminuric and microalbuminuric participants, and results were reported separately for each; however, the test/control diets did not specify what type of red meat was included, where applicable, so could not confirm beef inclusion. |
| Moderate Consumption of Red Meat, Compared to Soy or Non-Soy Legume, Has No Adverse Effect on Cardio-Metabolic Factors in Patients with Type 2 Diabetes. | Hassanzadeh-Rostami Z, Hemmatdar Z, Pishdad GR, Faghih S. | “Red meat” was not clearly defined such that beef may not have been the exclusive source of “red meat.” |
| Partial substitution of carbohydrate intake with protein intake from lean red meat lowers blood pressure in hypertensive persons. | Hodgson JM, Burke V, Beilin LJ, Puddey IB. | “Red meat” was not clearly defined such that beef may not have been the exclusive source of “red meat.” |
| Substitution of red meat with legumes in the therapeutic lifestyle change diet based on dietary advice improves cardiometabolic risk factors in overweight type 2 diabetes patients: a cross-over randomized clinical trial. | Hosseinpour-Niazi S, Mirmiran P, Hedayati M, Azizi F. | “Red meat” was not clearly defined such that beef may not have been the exclusive source of “red meat.” |
| Incorporation of lean red meat into a National Cholesterol Education Program Step I diet: a long-term, randomized clinical trial in free-living persons with hypercholesterolemia. | Hunninghake DB, Maki KC, Kwiterovich PO, Jr., Davidson MH, Dicklin MR, Kafonek SD. I | “Red meat” consisted mainly of veal, pork, and beef; beef was not exclusive source. |
| Assessment of Vascular Function in Response to High-Fat and Low-Fat Ground Beef Consumption in Men. | Lytle JR, Stanelle ST, Martin SE, Smith SB, Smith DR, Crouse SF. | Test diets varied in beef fat, not total beef intake |
| Changes in atherogenic dyslipidemia induced by carbohydrate restriction in men are dependent on dietary protein source | Mangravite LM, Chiu S, Wojnoonski K, Rawlings RS, Bergeron N, Krauss RM. | Similar beef protein content among treatment diets based on dietary composition table. |
| A single daily dose of soybean phytosterols in ground beef decreases serum total cholesterol and LDL cholesterol in young, mildly hypercholesterolemic men. | Matvienko OA, Lewis DS, Swanson M, Arndt B, Rainwater DL, Stewart J, Alekel DL. | Test diets contained the same amount of beef and only differed in supplemental soy isoflavone content |
| Bison meat has a lower atherogenic risk than beef in healthy men. | McDaniel J, Askew W, Bennett D, Mihalopoulos J, Anantharaman S, Fjeldstad AS, Rule DC, Nanjee NM, Harris RA, Richardson RS. | Only report % change in blood lipids (TC, LDL, HDL, TG) |
| Higher protein intake during resistance training does not potentiate strength, but modulates gut microbiota, in middle-aged adults: a randomized control trial. | McKenna CF, Salvador AF, Hughes RL, Scaroni SE, Alamilla RA, Askow AT, Paluska SA, Dilger AC, Holscher HD, De Lisio M, et al. | Diet included ~½ protein from beef and ~½ protein from beef protein isolate beverage |
| Therapeutic lifestyle change diet enriched in legumes reduces oxidative stress in overweight type 2 diabetic patients: a crossover randomised clinical trial. | Mirmiran P, Hosseinpour-Niazi S, Azizi F. | Type of red meat not specified |
| An oily fish diet increases insulin sensitivity compared to a red meat diet in young iron-deficient women. | Navas-Carretero S, Pérez-Granados AM, Schoppen S, Vaquero MP. | Type of red meat not specified |
| Low-sodium Dietary Approaches to Stop Hypertension-type diet including lean red meat lowers blood pressure in postmenopausal women. | Nowson CA, Wattanapenpaiboon N, Pachett A. | Diet included beef, veal and lamb |
| Human plasma lipid responses to red meat, poultry, fish, and eggs. | O'Brien BC, Reiser R. | Diet included beef, pork, and lamb |
| Controlled study of the effects of dietary protein on blood pressure in normotensive humans. | Prescott SL, Jenner DA, Beilin LJ, Margetts BM, Vandongen R. | Diet included mix of meats, not beef alone |
| A randomized controlled trial of the effect on blood pressure of dietary non-meat protein versus meat protein in normotensive omnivores. | Prescott SL, Jenner DA, Beilin LJ, Margetts BM, Vandongen R. | Diet included mix of meats, not beef alone |
| A dietary approach to prevent hypertension: a review of the Dietary Approaches to Stop Hypertension (DASH) Study. | Sacks FM, Appel LJ, Moore TJ, Obarzanek E, Vollmer WM, Svetkey LP, Bray GA, Vogt TM, Cutler JA, Windhauser MM, et al. | Multiple diet changes (DASH – also altered fruits, veg, cereals, etc.) |
| Varying dietary fat type of reduced-fat diets has little effect on the susceptibility of LDL to oxidative modification in moderately hypercholesterolemic subjects. | Schwab US, Vogel S, Lammi-Keefe CJ, Ordovas JM, Schaefer EJ, Li Z, Ausman LM, Gualtieri L, Goldin BR, Furr HC, et al. | Diet included beef tallow, not meat |
| Increased beef consumption increases apolipoprotein A-I but not serum cholesterol of mildly hypercholesterolemic men with different levels of habitual beef intake. | Smith DR, Wood R, Tseng S, Smith SB. | Compared Wagyu to commercial beef |
| Changes in Lipids and Inflammatory Markers after Consuming Diets High in Red Meat or Dairy for Four Weeks. | Turner KM, Keogh JB, Meikle PJ, Clifton PM. | Type of red meat not specified  No outcomes of interest |
| Partly replacing meat protein with soy protein alters insulin resistance and blood lipids in postmenopausal women with abdominal obesity. | van Nielen M, Feskens EJ, Rietman A, Siebelink E, Mensink M. | Diet included pork and beef |
| Effects of Adding Lean Red Meat to a U.S.-Style Healthy Vegetarian Dietary Pattern on Gut Microbiota and Cardiovascular Risk Factors in Young Adults: a Crossover Randomized Controlled Trial | Wang Y, Lindemann SR, Cross TL, Tang M, Clark CM, Campbell WW. | Diet included pork and beef |
| Plasma lipoprotein response to substituting fish for red meat in the diet. | Wolmarans P, Benadé AJ, Kotze TJ, Daubitzer AK, Marais MP, Laubscher R. | Diet included mutton and beef |
| The influence of consuming fatty fish instead of red meat on plasma levels of vitamins A, C and E. | Wolmarans P, Labadarios D, Benadé AJ, Kotze TJ, Louw ME. | Diet included mutton and beef |
| Effects of a prudent diet containing either lean beef and mutton or fish and skinless chicken on the plasma lipoproteins and fatty acid composition of triacylglycerol and cholesteryl ester of hypercholesterolemic subjects. | Wolmarans P, Laubscher JA, van der Merwe S, Kriek JA, Lombard CJ, Marais M, Vorster HH, Tichelaar HY, Dhansay MA, Benadé AJ. | Diet included mutton and beef |
| Effect of Low-Energy Diets Differing in Fiber, Red Meat, and Coffee Intake on Cardiac Autonomic Function in Obese Individuals With Type 2 Diabetes | Ziegler D, Strom A, Nowotny B, Zahiragic L, Nowotny PJ, Carstensen-Kirberg M, Herder C, Roden | Multiple diet changes (coffee and fiber) – unable to determine effect of red meat or beef alone  No outcomes of interest |
| Baseline Insulin Resistance Is a Determinant of the Small, Dense Low-Density Lipoprotein Response to Diets Differing in Saturated Fat, Protein, and Carbohydrate Contents | Wu X, Roussell MA, Hill AM, Kris-Etherton PM, Walzem RL. | Secondary analysis of Roussell paper (J Hum Hypertens, 2014)  Includes only measures of LDL and HDL fractions |

**Supplemental Table 3**. Risk of bias assessment

| **Author** | **Outcome** | **Overall Bias** |
| --- | --- | --- |
| Beauchesne-Rondeau 2003 | Blood lipids/Blood pressure | Low |
| Clina 2023 | Blood lipids/Blood pressure | Some concerns |
| de Mello 2006 | Blood lipids/Blood pressure | Low |
| Fleming 2021 | Blood lipids/Blood pressure | Low |
| Flynn 1981 | Blood lipids/Blood pressure | Some concerns |
| Flynn 1982 | Blood lipids/Blood pressure | Some concerns |
| Haub 2005 | Blood lipids/Blood pressure | Low |
| Hill 2015 | Blood lipids/Blood pressure | Low |
| Leaf 2009 | Blood lipids/Blood pressure | Some concerns |
| Magkos 2022 | Blood lipids/Blood pressure | Low |
| Mahon 2007 | Blood lipids/Blood pressure | Low |
| Maki 2020 | Blood lipids/Blood pressure | Low |
| Melanson 2003 | Blood lipids/Blood pressure | Low |
| Poddar 2013 | Blood lipids/Blood pressure | High |
| Roussell 2014 | Blood lipids/Blood pressure | Low |
| Roussell 2012 | Blood lipids/Blood pressure | Low |
| Santaliestra-Pasias 2022 | Blood lipids/Blood pressure | High |
| Scott 1994 | Blood lipids/Blood pressure | Some concerns |
| Turner 2016 | Blood lipids/Blood pressure | Some concerns |


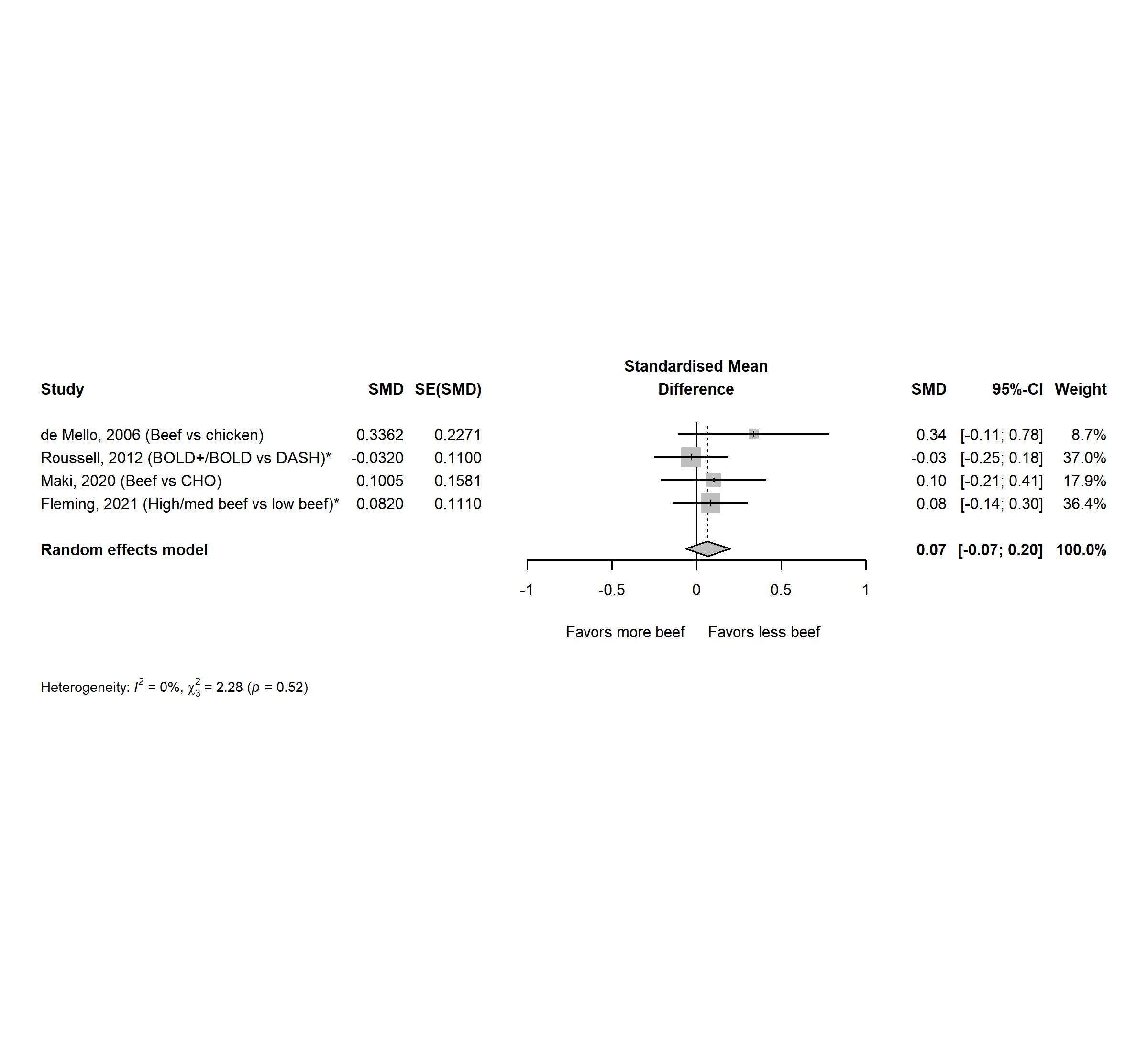


**Supplemental Figure S1:** Effect of higher beef intake on non-high density lipoprotein cholesterol (non-HDL-C). Values are standardized mean differences (SMD) of non-HDL-C between the beef diet and diets with less or no beef. Pooled effect p = 0.33. Abbreviations: BOLD = Beef in an Optimal Lean Diet, CHO = carbohydrate, DASH = Dietary Approaches to Stop Hypertension


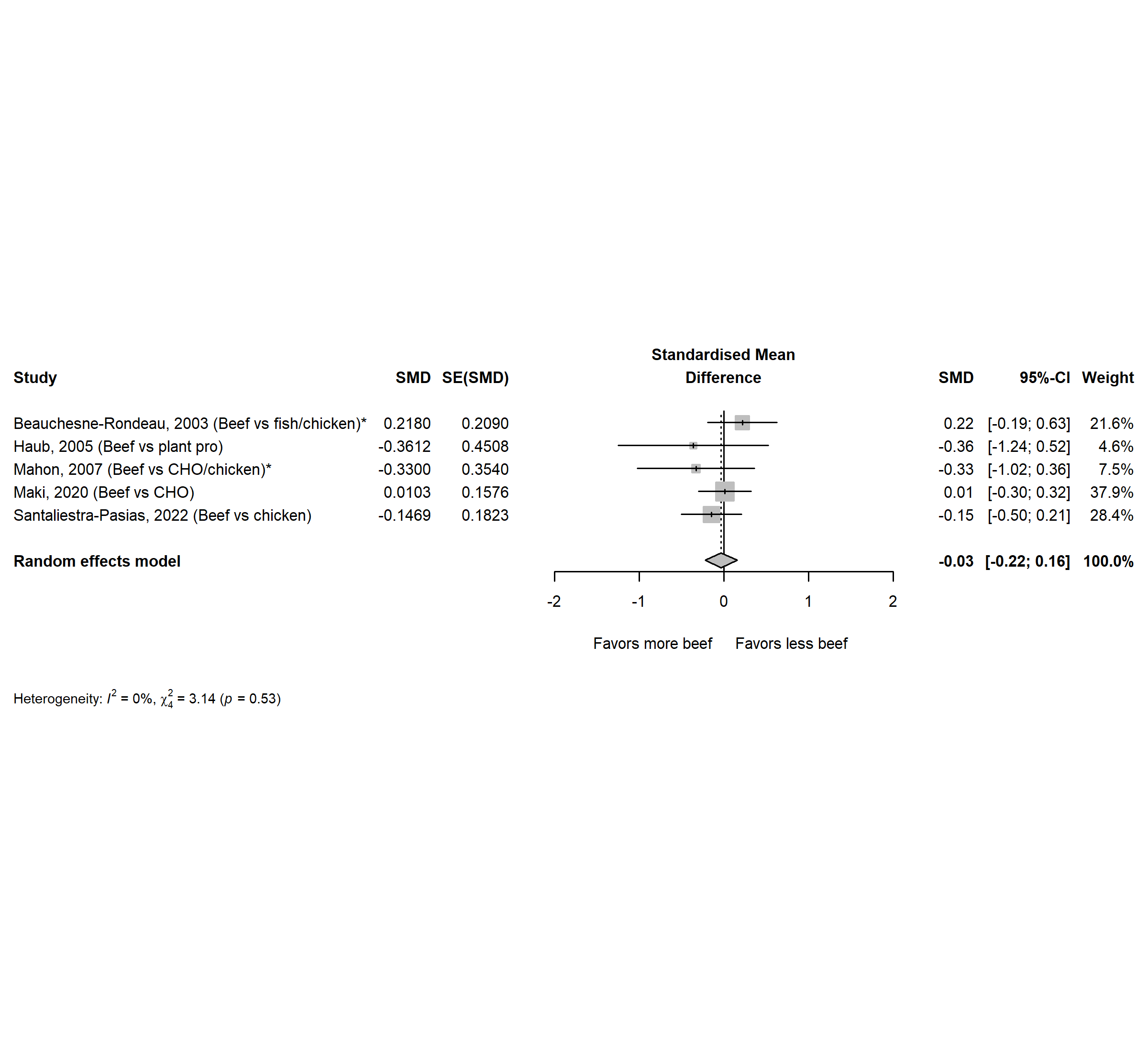


**Supplemental Figure S2:** Effect of higher beef intake on total/HDL-C ratio. Values are standardized mean differences (SMD) of total/HDL-C between the beef diet and diets with less or no beef. Pooled effect p = 0.74. Abbreviations: CHO = carbohydrate


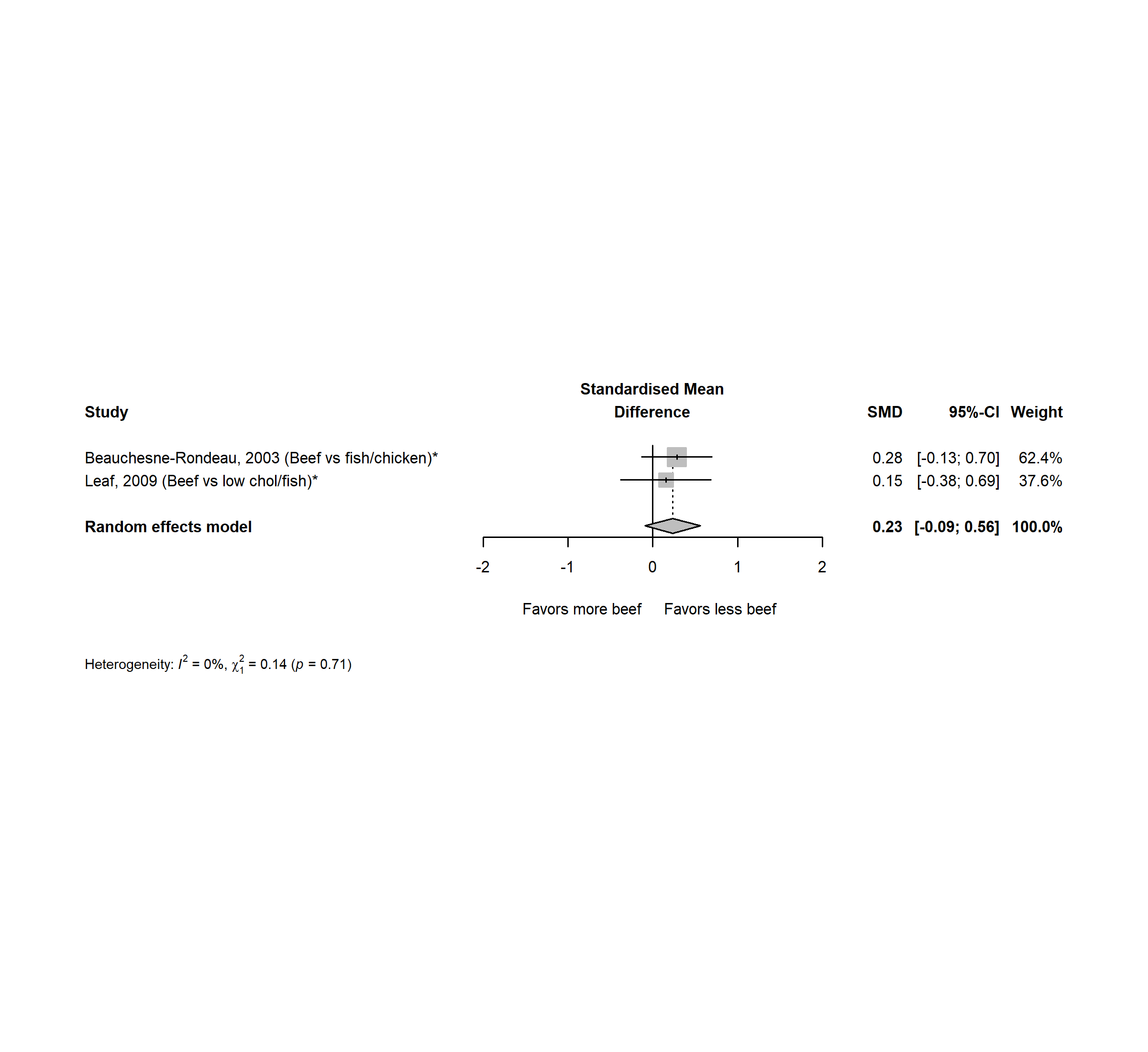


**Supplemental Figure S3:** Effect of higher beef intake on very low-density cholesterol (VLDL-C). Values are standardized mean differences (SMD) of VLDL-C between the beef diet and diets with less or no beef. Pooled effect p = 0.16. Each study included two comparisons which were pooled prior to running the model.


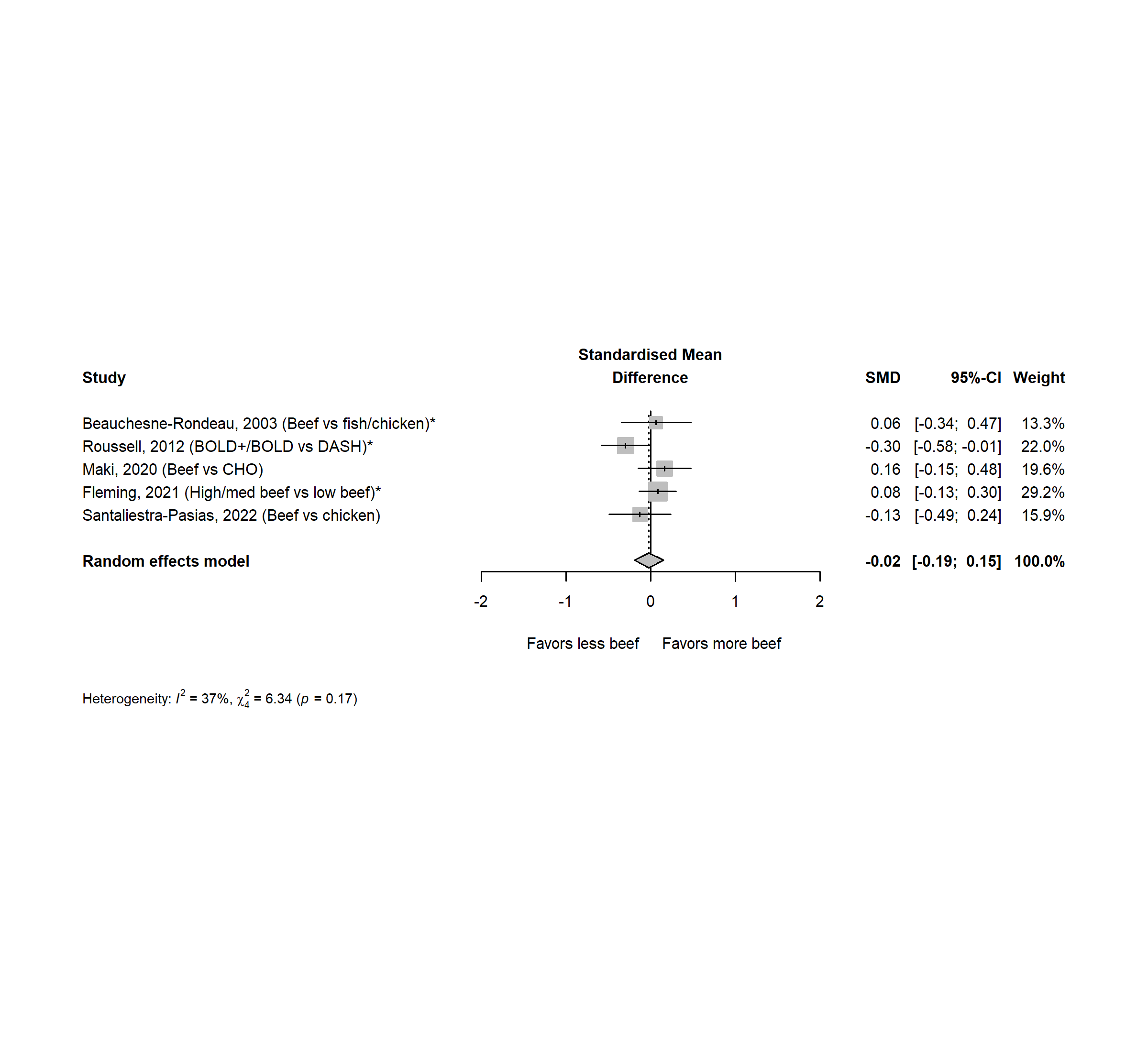


**Supplemental Figure S4:** Effect of higher beef intake on apolipoprotein A1 (apo A1). Values are standardized mean differences (SMD) of apo A1 between the beef diet and diets with less or no beef. Pooled effect p = 0.82. Abbreviations: BOLD = Beef in an Optimal Lean Diet, CHO = carbohydrate, DASH = Dietary Approaches to Stop Hypertension


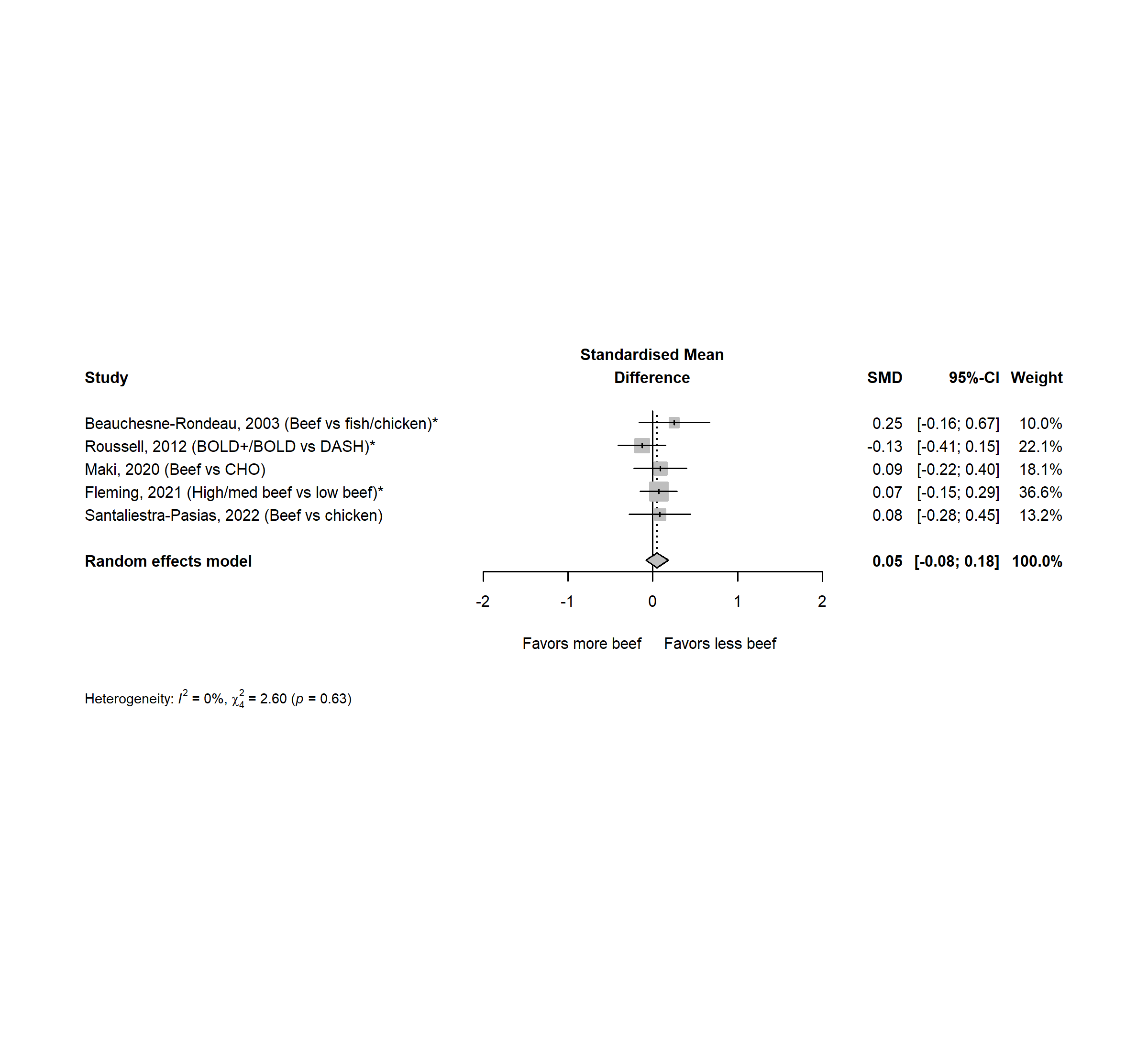


**Supplemental Figure S5:** Effect of higher beef intake on apolipoprotein B (apo B). Values are standardized mean differences (SMD) of apo B between the beef diet and diets with less or no beef. Pooled effect p = 0.46. Abbreviations: BOLD = Beef in an Optimal Lean Diet, CHO = carbohydrate, DASH = Dietary Approaches to Stop Hypertension


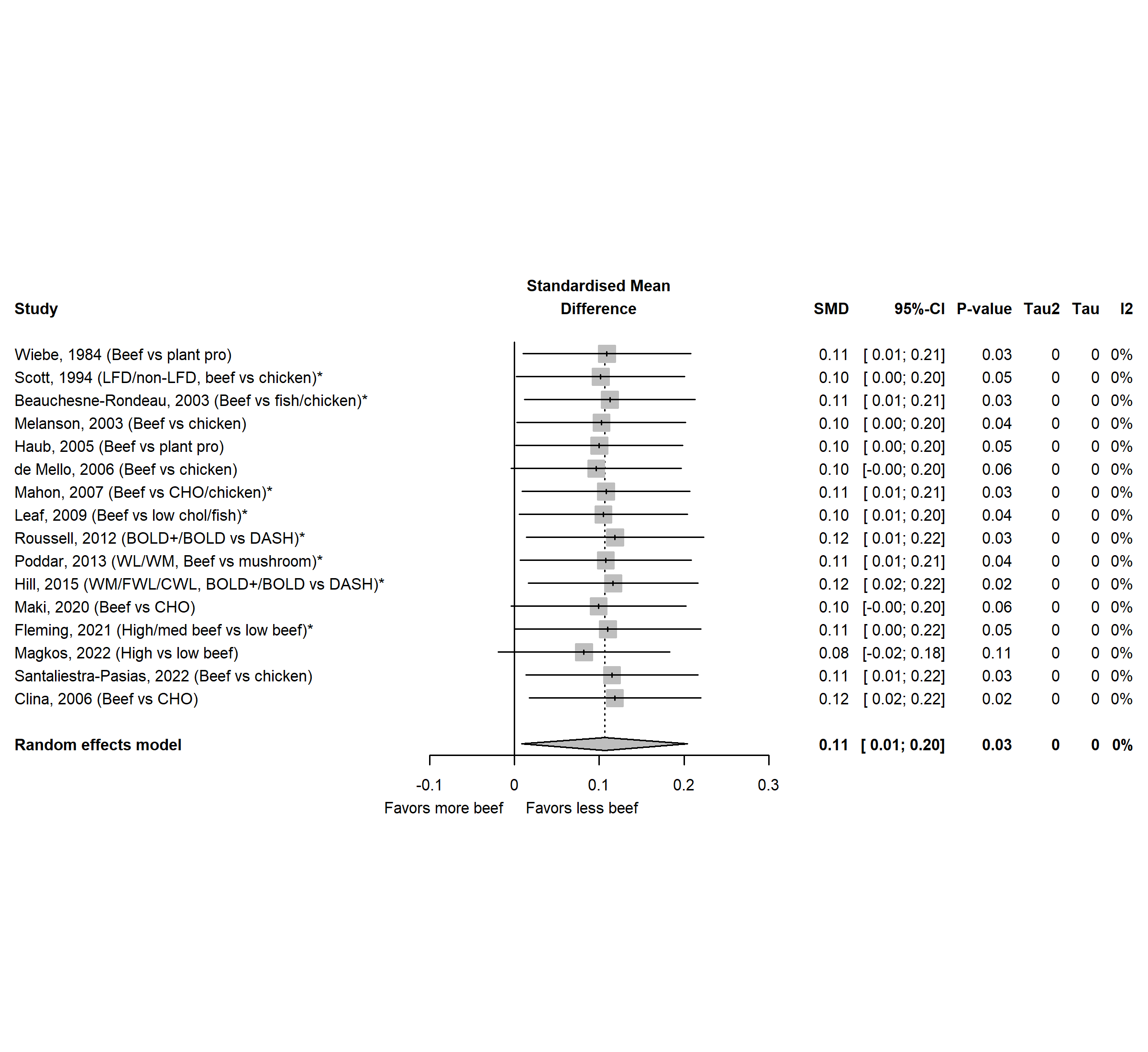


**Supplemental Figure S6:** Leave one study out sensitivity analysis for LDL-C. Values are standardized mean differences (SMD) of LDL-C between the beef diet and diets with less or no beef with the study excluded. Abbreviations: BOLD = Beef in an Optimal Lean Diet, CHO = carbohydrate, CWL = controlled weight loss, DASH = Dietary Approaches to Stop Hypertension, FWL = free living weight loss, LFD = low fat diet, WL = weight loss, WM = weight maintenance


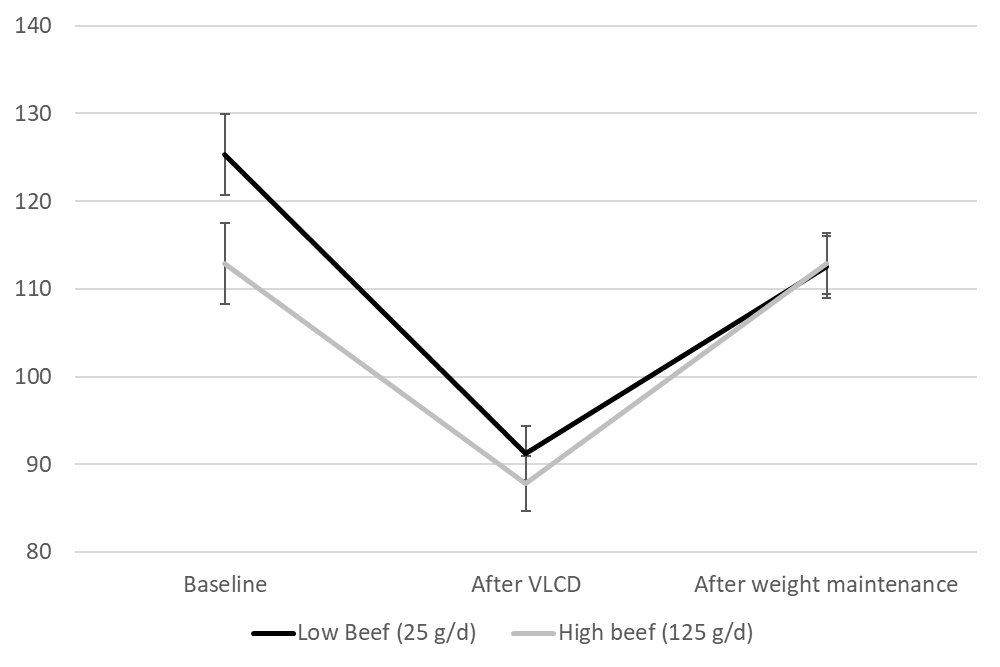


**Supplemental Figure S7:** Changes in LDL-C (intention-to-treat analysis) after an 8 week very low calorie diet (VLCD) followed by a low beef (25 g/d) or high beef (125 g/d) weight maintenance diet. From Magkos et al. (1)


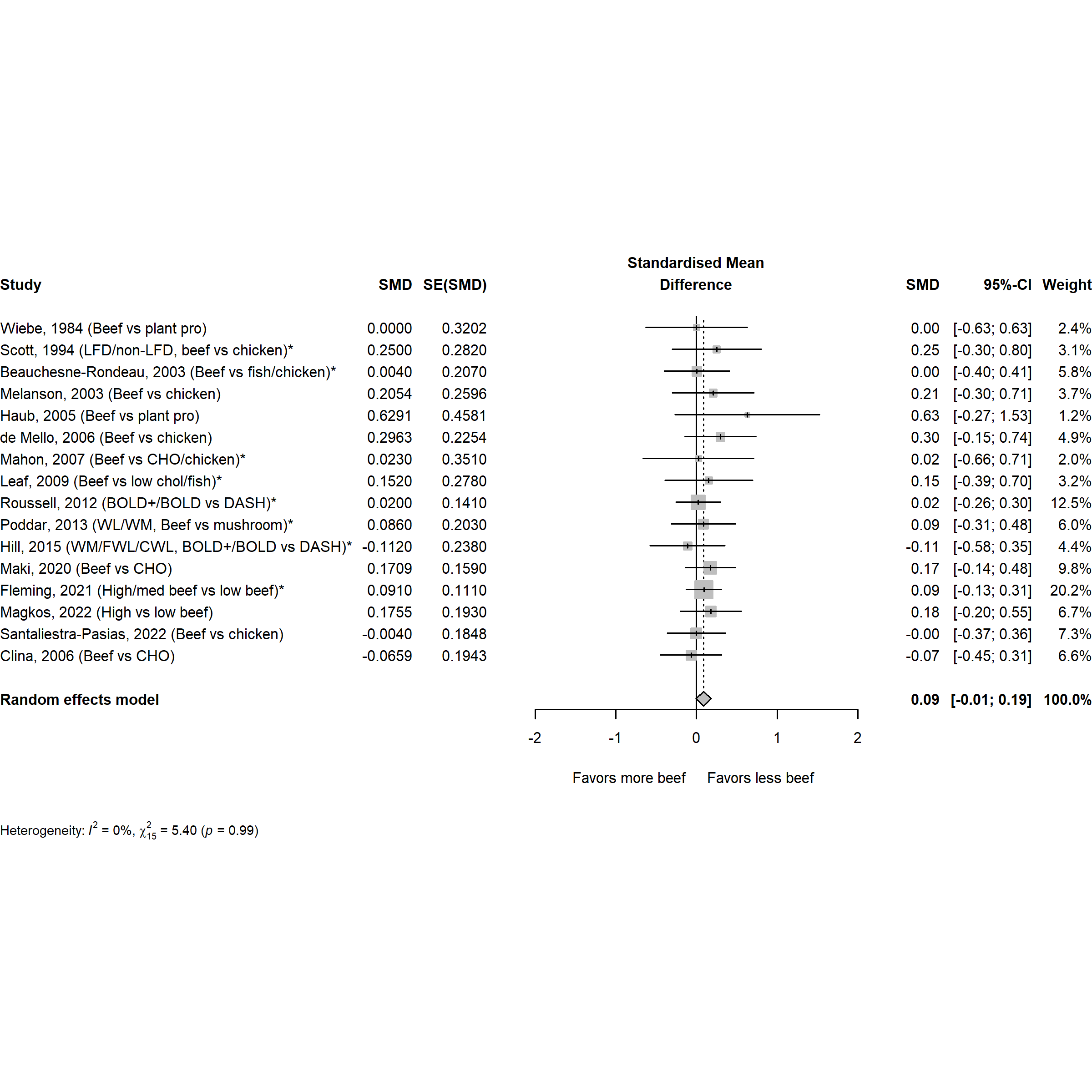


**Supplemental Figure S8:** Post hoc sensitivity analysis for LDL-C using end of weight loss lead in values for baseline for Magkos, 2022. Values are standardized mean differences (SMD) of LDL-C between the beef diet and diets with less or no beef. Pooled effect p = 0.08. Abbreviations: BOLD = Beef in an Optimal Lean Diet, CHO = carbohydrate, CWL = controlled weight loss, DASH = Dietary Approaches to Stop Hypertension, FWL = free living weight loss, LFD = low fat diet, WL = weight loss, WM = weight maintenance


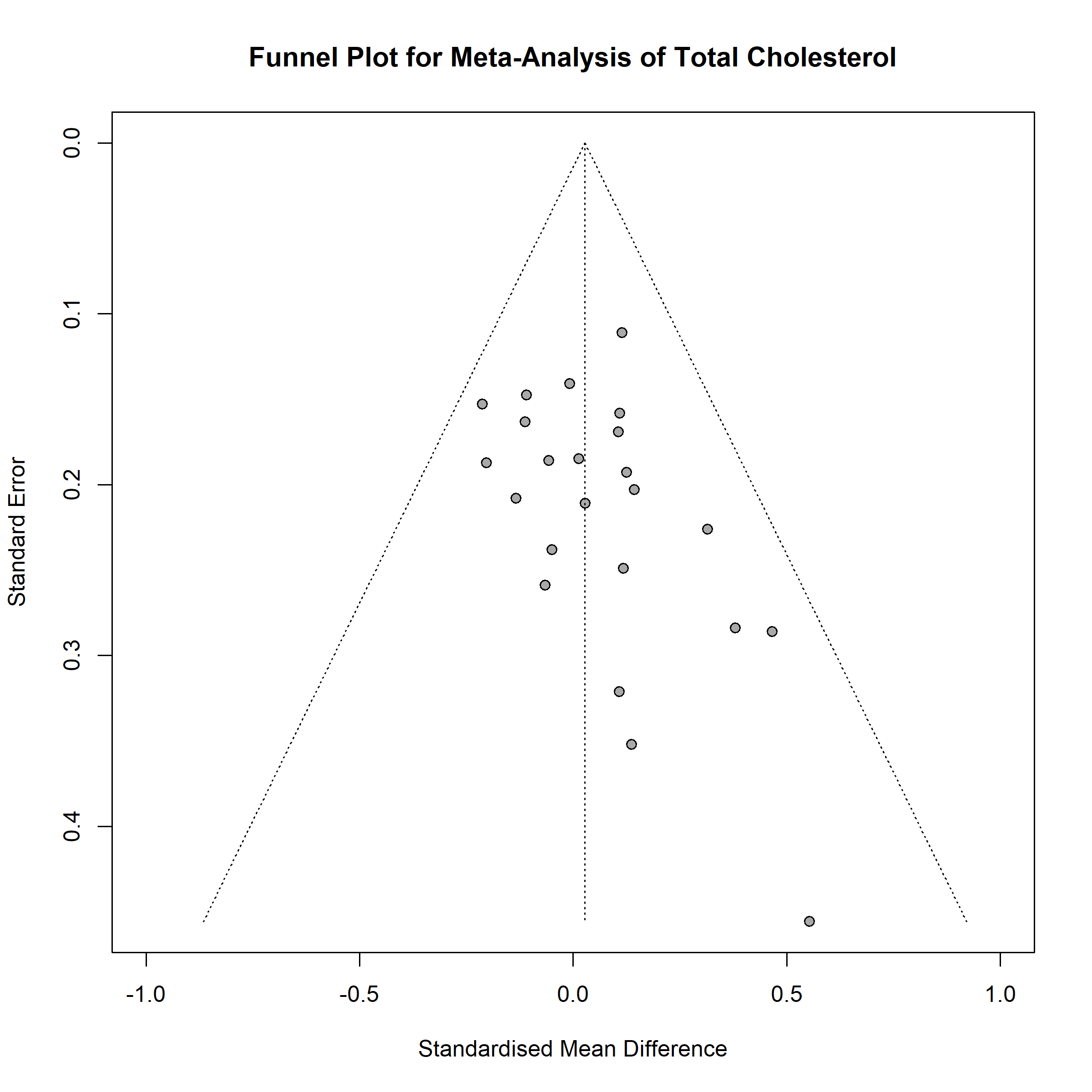


| Test for Small-Study Effects (Asymmetry in Funnel Plot): Total Cholesterol | | | | |
| --- | --- | --- | --- | --- |
| Test | Intercept | CI | t | p |
| Egger's | 1.0834 | -0.0205, 2.1873 | 1.9236 | 0.0681 |

**Supplemental Figure S9**: Funnel plot and Egger’s test assessing publication bias for total cholesterol.


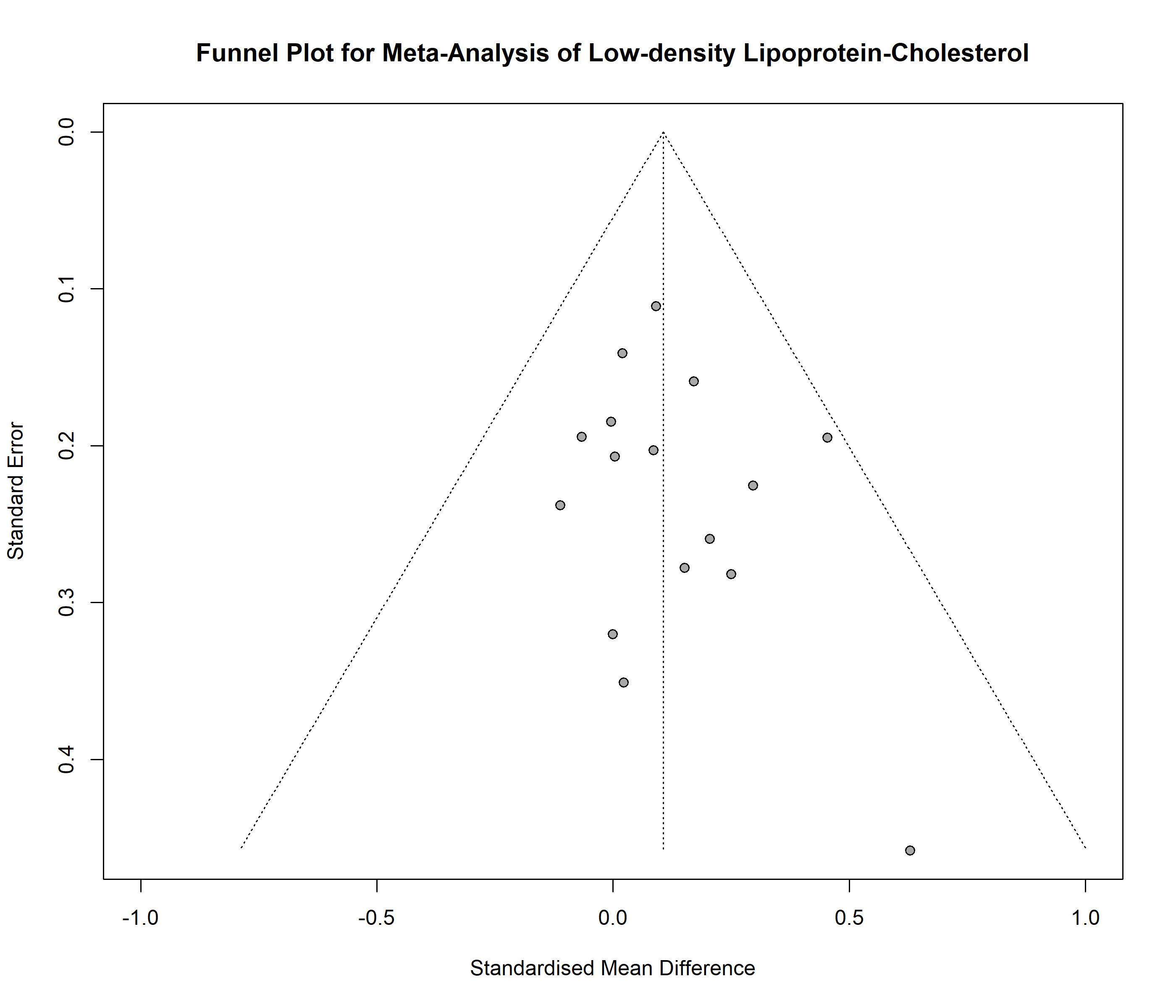


| Test for Small-Study Effects (Asymmetry in Funnel Plot): Low-density Lipoprotein-Cholesterol | | | | |
| --- | --- | --- | --- | --- |
| Test | Intercept | CI | t | p |
| Egger's | 0.5289 | -0.6018, 1.6596 | 0.9167 | 0.3748 |

**Supplemental Figure S10**: Funnel plot and Egger’s test assessing publication bias for LDL-C.


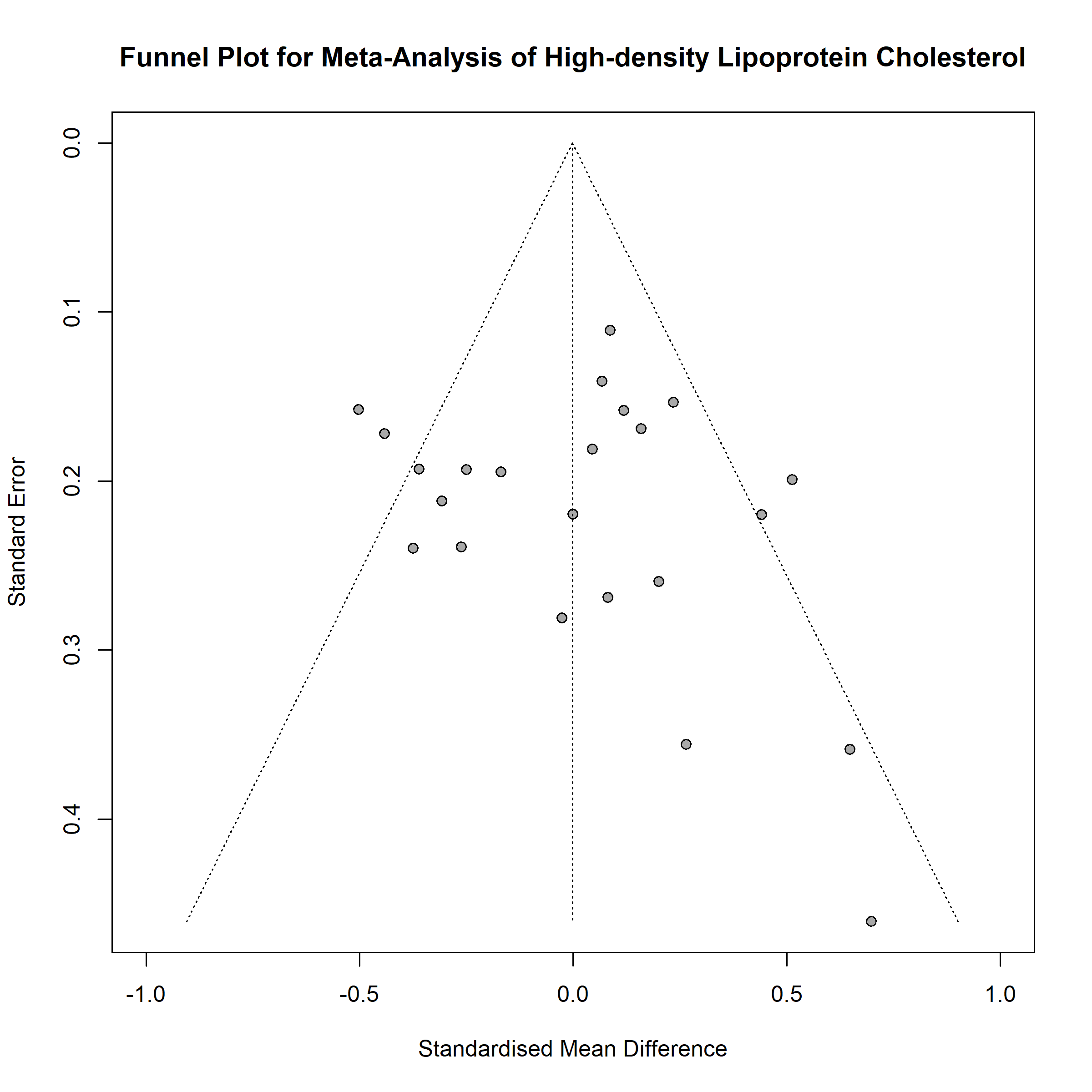


| Test for Small-Study Effects (Asymmetry in Funnel Plot): High-density Lipoprotein Cholesterol | | | | |
| --- | --- | --- | --- | --- |
| Test | Intercept | CI | t | p |
| Egger's | 0.7633 | -1.3826, 2.9093 | 0.6972 | 0.4933 |

**Supplemental Figure S11**: Funnel plot and Egger’s test assessing publication bias for HDL-C.


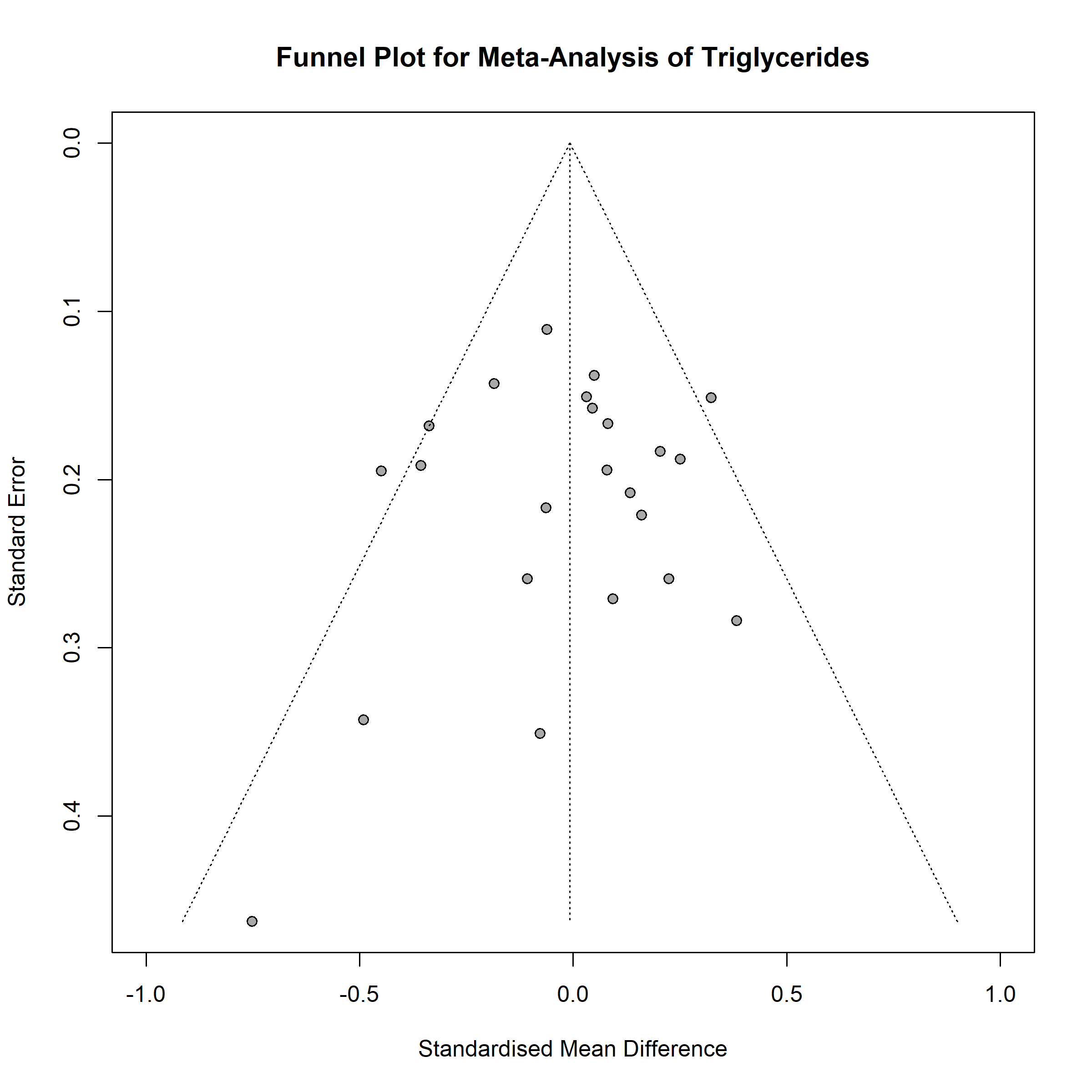


| Test for Small-Study Effects (Asymmetry in Funnel Plot): Triglycerides | | | | |
| --- | --- | --- | --- | --- |
| Test | Intercept | CI | t | p |
| Egger's | -0.4292 | -2.1111, 1.2528 | -0.5001 | 0.6222 |

**Supplemental Figure S12:** Funnel plot and Egger’s test assessing publication bias for TG.


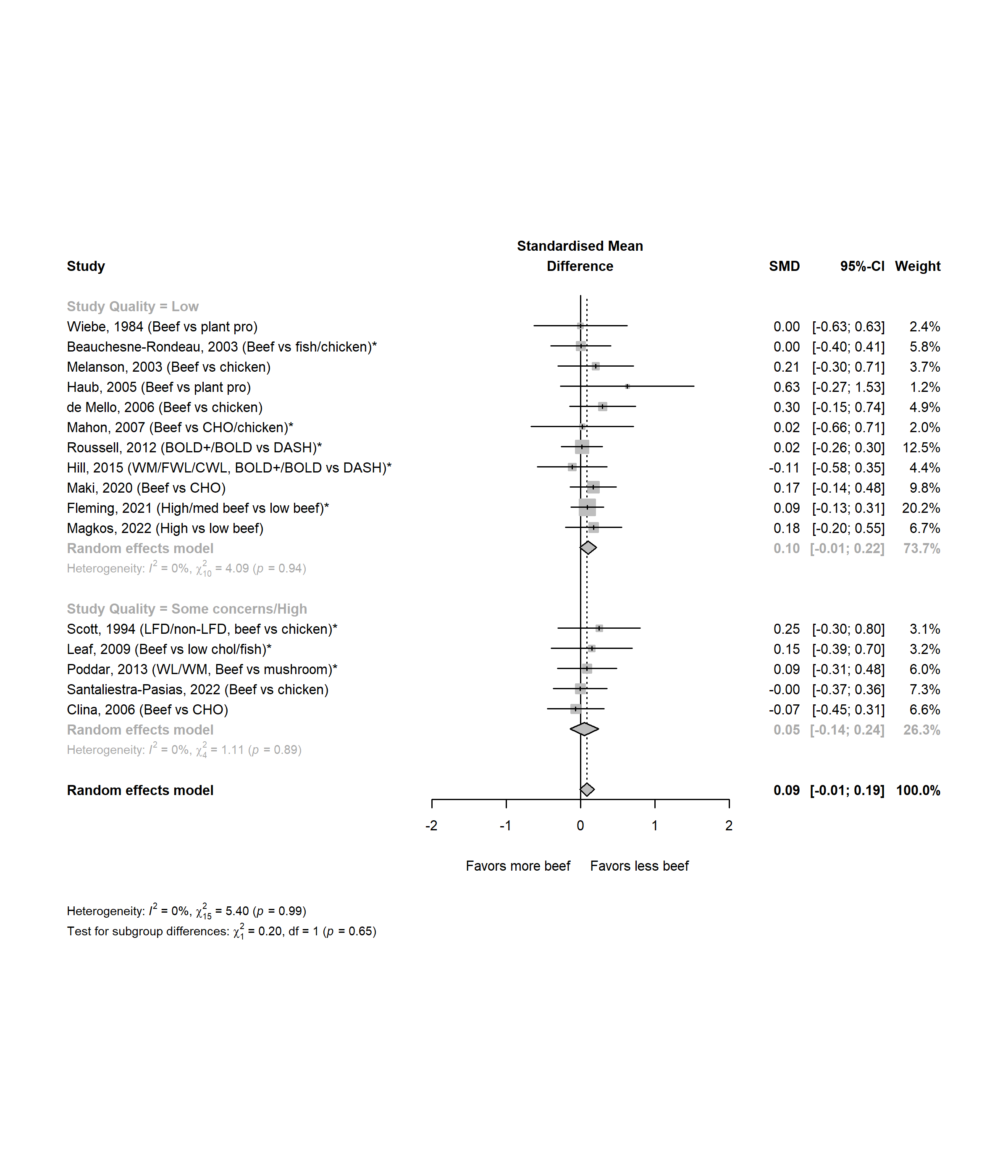


**Supplemental Figure S13:** Post-hoc subgroup analysis by study quality for LDL-C using end of non-beef weight loss lead in values for baseline for (1). Values are standardized mean differences (SMD) of LDL-C between the beef diet and diets with less or no beef. Pooled effect for low risk of bias p = 0.08. Pooled effect for some concerns/high risk of bias p = 0.60. Abbreviations: BOLD = Beef in an Optimal Lean Diet, CHO = carbohydrate, CWL = controlled weight loss, DASH = Dietary Approaches to Stop Hypertension, FWL = free living weight loss, LFD = low fat diet, WL = weight loss, WM = weight maintenance

**References**

1. Magkos F, Rasmussen SI, Hjorth MF, Asping S, Rosenkrans MI, Sjödin AM, Astrup AV, Geiker NRW. Unprocessed red meat in the dietary treatment of obesity: a randomized controlled trial of beef supplementation during weight maintenance after successful weight loss. *Am J Clin Nutr.* 2022 Dec 19;116:1820-30.
